# Crystal structures of SAMHD1 inhibitor complexes reveal the mechanism of water-mediated dNTP hydrolysis

**DOI:** 10.1038/s41467-020-16983-2

**Published:** 2020-06-23

**Authors:** Elizabeth R. Morris, Sarah J. Caswell, Simone Kunzelmann, Laurence H. Arnold, Andrew G. Purkiss, Geoff Kelly, Ian A. Taylor

**Affiliations:** 1 Macromolecular Structure Laboratory, The Francis Crick Institute, 1 Midland Road, London, NW1 1AT UK; 2 Structural Biology Science Technology Platform, The Francis Crick Institute, 1 Midland Road, London, NW1 1AT UK; 3 The Medical Research Council Biomedical NMR Centre, The Francis Crick Institute, 1 Midland Road, London, NW1 1AT UK; 4 Present Address: AstraZeneca, 50F49, Mereside, Alderley Park, Macclesfield, Cheshire SK10 4TG UK; 5 Present Address: Pelago Bioscience, Banvaktsvägen 20, 171 48 Solna, Sweden

**Keywords:** Enzyme mechanisms, Structural biology, X-ray crystallography

## Abstract

SAMHD1 regulates cellular 2′-deoxynucleoside-5′-triphosphate (dNTP) homeostasis by catalysing the hydrolysis of dNTPs into 2′-deoxynucleosides and triphosphate. In CD4^+^ myeloid lineage and resting T-cells, SAMHD1 blocks HIV-1 and other viral infections by depletion of the dNTP pool to a level that cannot support replication. SAMHD1 mutations are associated with the autoimmune disease Aicardi–Goutières syndrome and hypermutated cancers. Furthermore, SAMHD1 sensitises cancer cells to nucleoside-analogue anti-cancer therapies and is linked with DNA repair and suppression of the interferon response to cytosolic nucleic acids. Nevertheless, despite its requirement in these processes, the fundamental mechanism of SAMHD1-catalysed dNTP hydrolysis remained unknown. Here, we present structural and enzymological data showing that SAMHD1 utilises an active site, bi-metallic iron-magnesium centre that positions a hydroxide nucleophile in-line with the P^α^-O^5′^ bond to catalyse phosphoester bond hydrolysis. This precise molecular mechanism for SAMHD1 catalysis, reveals how SAMHD1 down-regulates cellular dNTP and modulates the efficacy of nucleoside-based anti-cancer and anti-viral therapies.

## Abstract (web summary)

SAMHD1 catalyses the hydrolysis of dNTPs into 2′-deoxynucleosides and triphosphate and is an important regulator of cellular dNTP homeostasis. Here, the authors provide insights into the catalytic mechanism of SAMHD1 by performing kinetic measurements and determining crystal structures of α-β-imido-dNTP inhibitor complexes, which reveal a bi-metallic iron-magnesium centre and catalytic hydroxyl molecule in the active site of the enzyme.

## Introduction

Sterile alpha motif and HD domain-containing protein 1 (SAMHD1) is an important regulator of cellular 2′-deoxynucleoside-5′-triphosphate (dNTP) in mammalian cells^1^. SAMHD1 is widely expressed in most tissue types^2,3^ and catalyses the hydrolysis of dNTPs into their constituent triphosphate and 2′-deoxynucleoside^4,5^. HIV-1 and other retroviruses replicate poorly in terminally differentiated cells of the myeloid lineage and resting T-cells^6–12^. This is a result of SAMHD1-dNTP triphosphohydrolase activity that blocks early stage infection through depletion of the dNTP pool to a level that cannot support viral reverse transcription^13–16^. In addition, SAMHD1 also inhibits replication of the DNA viruses, herpes simplex virus type 1 and vaccinia virus in monocyte-derived macrophages^17,18^, and hepatitis B virus in hepatic cells^19^. In HIV-2 and related simian viruses (SIVs), this restriction is overcome by the lentiviral accessory protein Vpx that targets human SAMHD1 for degradation by the proteasomal pathway^9,11,12^ through interactions with the cullin-4 ligase substrate adaptor DCAF1^20–22^.

In addition to restriction of HIV-1 replication, SAMHD1 is an interferon-induced gene^2,23^ and regulator of the innate immune response^24,25^. Germ line mutations in SAMHD1 are associated with early onset stroke^26^ and the rare hereditary autoimmune disease Aicardi–Goutières syndrome, which mimics congenital viral infection^25,27^. SAMHD1 mutations have also been identified as potential drivers of chronic lymphocytic leukaemia^28–30^ and are present in hypermutated cancers^31^. Conversely, high SAMHD1 expression in acute myelogenous leukaemia patients is associated with efficacy reduction of the nucleoside-analogue anti-cancer drugs Cytarabine and Clofarabine^32–34^ as a result of SAMHD1 hydrolysis of their triphosphorylated forms^35–38^. Therefore, modulation of SAMHD1 activity by drugs or potentially by Vpx targeting of SAMHD1 for proteasomal degradation has been proposed as a strategy for improving anti-cancer and anti-HIV therapies^32,36,39^. More recently, a role for SAMHD1 in the regulation of DNA repair at stalled replication forks and the suppression of the interferon response by cytosolic nucleic acids has been proposed^40,41^.

In humans, SAMHD1 is a 626-residue protein. It comprises an N-terminal nuclear localisation signal^42^ and two major structural domains: the N-terminal sterile alpha motif (SAM) domain, which is a helical scaffold for protein- or nucleic acid-binding in other SAM domain-containing proteins^43,44^, and the SAMHD1 HD phosphohydrolase domain, which is named after the two pairs of conserved histidine and aspartate residues that coordinate a metal ion at the active site^45^. In addition, residues in a C-terminal region are targeted by lentiviral Vpx accessory proteins to recruit SAMHD1 to the proteasome for degradation^22,46^.

Although the HD-domain contains the active site, dNTP hydrolysis requires assembly of SAMHD1 into homo-tetramers^47^. Here, sequences at the N and C termini of the HD-domain stabilise inter-monomer protein–protein interactions and incorporate four pairs of allosteric nucleotide-binding sites, AL1 and AL2, which regulate the enzyme through combined binding of G-based (AL1) and 2′-deoxynucleoside (AL2) triphosphates^4,48–50^. GTP is the physiological ligand for the first allosteric site, AL1, although dGTP can also coordinate this site^16,50^. The second allosteric site, AL2, is specific for a dNTP with the following preference order: dATP > dGTP > TTP > dCTP^51–54^ and a magnesium ion coordinates the triphosphates of each GTP-dNTP pair in adjacent AL1 and AL2-binding sites^48,49,55^. In addition, SAMHD1 activity is cell cycle regulated by CyclinA2/CDK2 phosphorylation at Threonine 592^56–58^ through effects on tetramer stability that modulate activity^16^. Removal of regulation through T592 dephosphorylation or mutation may enable SAMHD1 to inhibit HIV-1 in cycling cells^56^.

Given that SAMHD1 allosteric regulation, assembly and the coupling of tetramerisation to catalysis have been studied so extensively, it is surprising that the molecular details and mechanism of catalysis remain unknown. Because of the importance of SAMHD1 both as a potential therapeutic target and a key intracellular regulator of metabolism, we have investigated the catalytic mechanism and inhibition of SAMHD1 through combined structural and biochemical studies. We first employed dNTP analogues modified at the α,β-phosphoester bond to probe the enzymology of SAMHD1-dNTP triphosphohydrolase activity that cleaves the labile bond between the α-phosphorus and 5′ oxygen (P^α^-O^5′^) of a substrate dNTP. These data demonstrate the contribution of the chemical moieties that are proximal to the P^α^-O^5′^ labile bond and the role of water in the hydrolysis reaction. We next determined X-ray co-crystal structures of SAMHD1 with non-hydrolysable competitive inhibitor α,β-imido-dNTP analogues. These data reveal a previously unreported iron-magnesium bi-metallic centre at the SAMHD1-active site that positions a hydroxide nucleophile for in-line attack on the substrate dNTP P^α^-O^5′^ bond. Further structural and biophysical experiments demonstrate key residues that are important for metal binding and substrate binding at the active site and show that conserved catalytic residue Histidine 215 protonates the O^5′^ leaving group, to stabilise the negative charge that O^5′^ accumulates after adduction of water to the α-phosphate.

Together, our results now reveal the physiological binding mode of substrate dNTPs in the SAMHD1-active site, provide a chemical mechanism for SAMHD1-dNTP hydrolysis and explain the nature of SAMHD1 inhibition by α,β-imido-dNTP analogues. These observations contribute significantly to our understanding of SAMHD1 regulation of cellular dNTP, its effects on the efficacy of nucleoside-based anti-cancer and anti-viral therapies, and provide insight into the design of future SAMHD1-active site inhibitors.

## Results

### SAMHD1 and α-β-modified dNTP analogues

To identify dNTP analogues useful for structural studies of the catalytic mechanism of SAMHD1 triphosphohydrolase activity, we assembled a panel of α,β-imido (dNMPNPP) and α,β-methyleno (dNMPCPP) dNTP analogues (Supplementary Fig. 1). These analogues are modified at the bridging α,β-oxygen atom that is in close proximity to the P^α^-O^5′^ phosphoester bond that is cleaved by SAMHD1 in the triphosphohydrolysis reaction. The nucleotide analogues were tested for their properties as substrates, allosteric activators or inhibitors of SAMHD1 catalytic activity using both ^1^H NMR spectroscopy and a coupled enzyme assay to monitor dNTP triphosphohydrolase activity, and size exclusion chromatography with multi-angle laser light scattering (SEC-MALLS) to assess their effects on SAMHD1 tetramerisation.

**Fig. 1:**
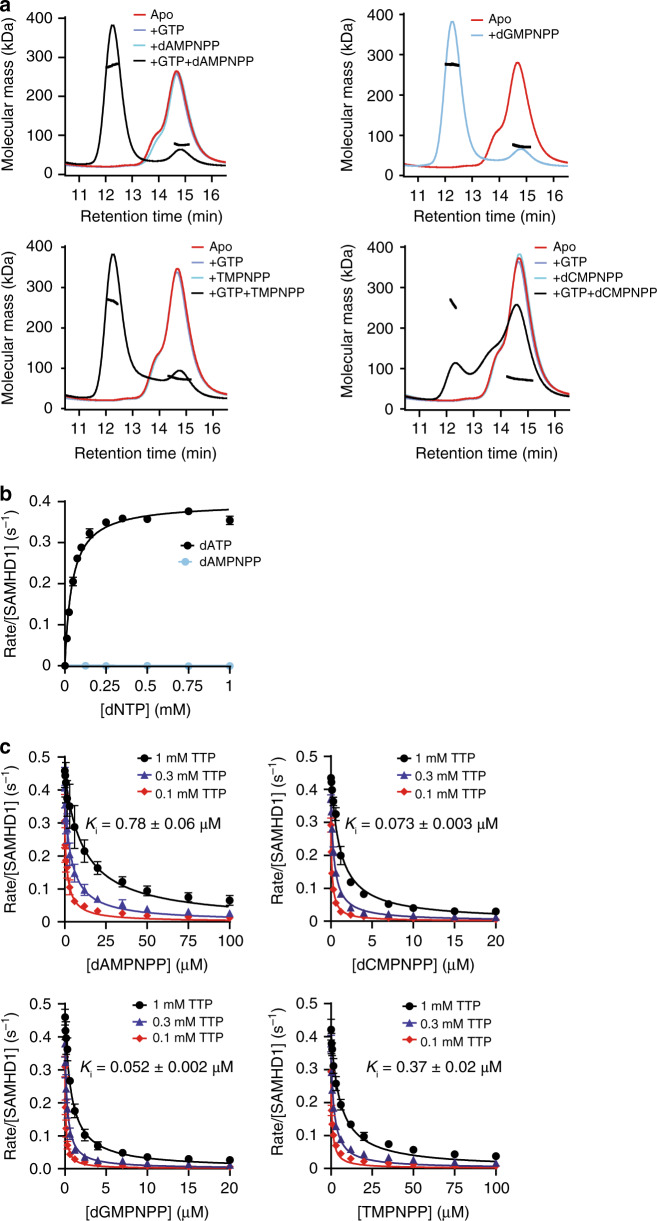
SAMHD1 tetramerisation and catalytic inhibition by dNMPNPP nucleotides. **a** SEC-MALLS analysis of SAMHD1 monomer–dimer–tetramer equilibrium upon addition of dNMPNPP nucleotides. In each panel, the solid lines are the chromatograms from the output of the differential refractometer and the black scatter points are the weight-averaged molar masses determined at 1-s intervals throughout elution of chromatographic peaks, SAMHD1 monomer–dimers elute at 14–16 min, tetramers at 12 min. In each panel, the curves shown are: (red) apo-SAMHD1; (blue) SAMHD1 and 0.2 mM GTP; (cyan) SAMHD1 and 0.5 mM indicated dNMPNPP analogue; and (black) SAMHD1, 0.2 mM GTP and 0.5 mM indicated dNMPNPP analogue. For dGMPNPP, which can induce tetramer formation in the absence of GTP, only the chromatograms for the apo (red) and after addition of dGMPNPP (cyan) are shown. **b** Steady-state kinetic analysis of GTP-stimulated hydrolysis of dATP and dAMPNPP by SAMHD1, measured using the MDCC-PBP florescence-based coupled SAMHD1-Ppx assay. The dependence of the rate on substrate concentration is plotted for (black) dATP and (cyan) dAMPNPP. For the dATP reaction the solid line is the best fit to the data using the Michaelis–Menten expression, that gives values for the derived constants *K*_M_ and *k*_cat_ of 44 ± 3 µM and 0.4 ± 0.01 s^−1^, respectively. Error bars represent the standard error of the mean (s.e.m.) of three independent measurements. **c** Determination of dNMPNPP inhibition constants (*K*_i_) using the florescence-based coupled assay. Plots show the dependence of the SAMHD1 hydrolysis rate of 0.1, 0.3 and 1 mM TTP on the concentration of each dNMPNPP. Reported *K*_i_ values (inset and Supplementary Table 1) were derived from global fitting of each 3-concentration dataset. Error bars represent the s.e.m. of three independent measurements. Source data for **b** and **c** are provided in the Source Data file.

SEC-MALLS analysis revealed that all four dNMPNPP analogues, which have an imido substitution at the α,β-bridging oxygen (Supplementary Fig. 1), when combined with GTP, support the formation of stable SAMHD1 tetramers, albeit to varying degrees (Fig. 1a). Near-complete tetramerisation was observed with dAMPNPP, while dCMPNPP promoted the least tetramerisation and dGMPNPP was able to support tetramerisation without additional GTP, presumably by occupying both AL1 and AL2 allosteric sites.

In enzymatic assays employing GTP as an AL1-activator, no hydrolysis of any dNMPNPP was observed, when monitored either by the coupled enzyme assay or by ^1^H NMR spectroscopy (Fig. 1b and Supplementary Fig. 2). Further investigation using inhibition assays revealed that all dNMPNPP analogues were actually competitive inhibitors of SAMHD1-dNTP triphosphohydrolase activity, with inhibition constants (*K*_i_) ranging from 0.78 µM for dAMPNPP to 0.052 µM for dGMPNPP, with the following rank order of increasing affinity: dAMPNPP < TMPNPP < dCMPNPP < dGMPNPP (Fig. 1c and Supplementary Table 1).

**Fig. 2:**
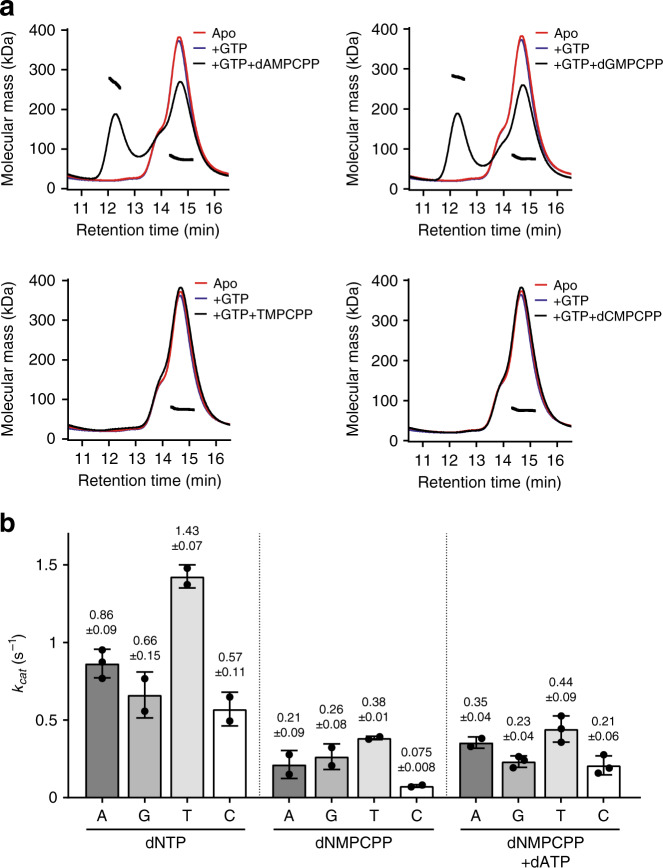
SAMHD1 tetramerisation and hydrolysis of dNMPCPP analogues. **a** SEC-MALLS analysis of SAMHD1 monomer–dimer–tetramer equilibrium upon addition of dNMPCPP nucleotides. In each panel, the solid lines are the chromatograms from the output of the differential refractometer and the black scatter points are the weight-averaged molar masses determined at 1-s intervals throughout elution of chromatographic peaks. In each panel, the displayed curves are: (red) apo-SAMHD1; (blue) SAMHD1 and 0.2 mM GTP; and (black) SAMHD1, 0.2 mM GTP and 0.5 mM indicated dNMPCPP analogue. **b** Bar chart of the apparent *k*_cat_ values derived from ^1^H NMR data recorded for GTP-stimulated SAMHD1 hydrolysis of each dNTP, dNMPCPP and dNMPCPP/dATP reaction, see Supplementary Fig. 3. Points overlaid in each bar are the individual data measurements, the error bars represent the standard deviation (s.d.) from at least two independent measurements. The source data are provided in the Source Data file.

By contrast, the dNMPCPP analogues, which have a methyleno substitution at the α,β-bridging oxygen (Supplementary Fig. 1) poorly stabilise the catalytically active SAMHD1 tetramer with only dAMPCPP and dGMPCPP, combined with GTP, supporting tetramerisation sufficiently well for SAMHD1 tetramers to be observed in SEC-MALLS experiments (Fig. 2a). However, assessment of dNMPCPP analogues as SAMHD1 substrates demonstrated that all are hydrolysed by GTP-activated SAMHD1, with apparent catalytic rate constants, *k*_cat_, ranging from 0.38 to 0.075 s^−1^, with the rank order of: TMPCPP > dAMPCPP ≈ dGMPCPP > dCMPCPP (Fig. 2b, Supplementary Fig. 3 and Supplementary Table 2). The *k*_cat_ parameters determined for dNMPCPP analogues were 3- to 8-fold lower than those determined for cognate dNTP substrates, which range from 1.43 to 0.57 s^−1^ and have the rank order: TTP > dATP > dGTP ≈ dCTP (Fig. 2b, Supplementary Fig. 3 and Supplementary Table 2). However, addition of low concentrations of dATP (10 µM) to dNMPCPP reactions, to induce tetramerisation through enhanced AL2 binding, increased the turnover of the dNMPCPP analogues with *k*_cat_ values now ranging from 0.44 to 0.21 s^−1^ and restored the rank order of turnover to that observed with the cognate dNTPs: TMPCPP > dAMPCPP > dGMPCPP ≈ dCMPCPP. The most significant effect of dATP addition was observed with dCMPCPP, with *k*_cat_ being increased 2.5-fold, presumably due to the limited ability of C-based nucleotides to induce SAMHD1 tetramerisation by AL2 binding. These data suggest that dNMPCPP analogues are actually SAMHD1 substrates with *k*_cat_ comparable to their analogous cognate dNTPs, but a weak association at AL2 and a lack of capacity to induce SAMHD1 tetramerisation results in decreased catalytic turnover of dNMPCPP at the active site.

Taking all these data into account, it is apparent that although the dNMPCPP analogues are SAMHD1 substrates, they are poor substitutes for cognate dNTPs because they are unable to induce tetramer assembly. By contrast, dNMPNPP analogues, while not SAMHD1 substrates, make excellent candidates for co-crystallisation, as they support SAMHD1 tetramerisation, through AL2 binding, coordinate the catalytic site with sub-µM affinities and are entirely refractory to hydrolysis. Therefore, we chose these analogues for structural studies aimed at understanding the molecular details of the SAMHD1 catalytic site.

### Crystal structures of SAMHD1–dNMPNPP inhibitor complexes

We co-crystallised the SAMHD1 catalytic domain (residues 109–626) that lacks the N-terminal SAM domain with dNMPNPP inhibitor analogues in the active site and additional nucleotides at allosteric sites to support tetramer assembly. These co-crystal structures reveal how a substrate dNTP, metal ions and water molecules are coordinated at the active site prior to catalysis.

Co-crystal structures were determined using either wild-type (wt) wt-SAMHD1(109-626) or a D137N-SAMHD1(109-626) AL1 allosteric site mutant that generally produced higher resolution diffraction than wt co-crystals. A further effect of the D137N substitution is that D137N-SAMHD1 is activated by xanthosine-5′-triphosphate (XTP) rather than GTP through AL1-binding but nevertheless maintains comparable catalytic parameters for dNTP hydrolysis as those observed for wt-SAMHD1 and GTP (Supplementary Fig. 4).

We first determined structures of two co-complexes of D137N-SAMHD1(109-626) in the presence of Mg^2+^, which is required for tetramer assembly and catalysis, with (i) XTP (AL1) and dAMPNPP (AL2 and active site), and (ii) XTP (AL1) and dGMPNPP (AL2 and active site). In addition, we determined the structures of two co-complexes of wt-SAMHD1(109-626), also in the presence of Mg^2+^, with (i) GTP (AL1), dATP (AL2) and dCMPNPP (active site) and (ii) GTP (AL1), dATP (AL2) and TMPNPP (active site). dATP was added to the latter two complexes to stimulate tetramer assembly, as dCMPNPP and TMPNPP are poor AL2 activators (Fig. 1a). The structures were solved by molecular replacement and details of the unit cell contents, data collection statistics, structure determination and model refinements are presented in Supplementary Tables 3 and 4.

**Fig. 3:**
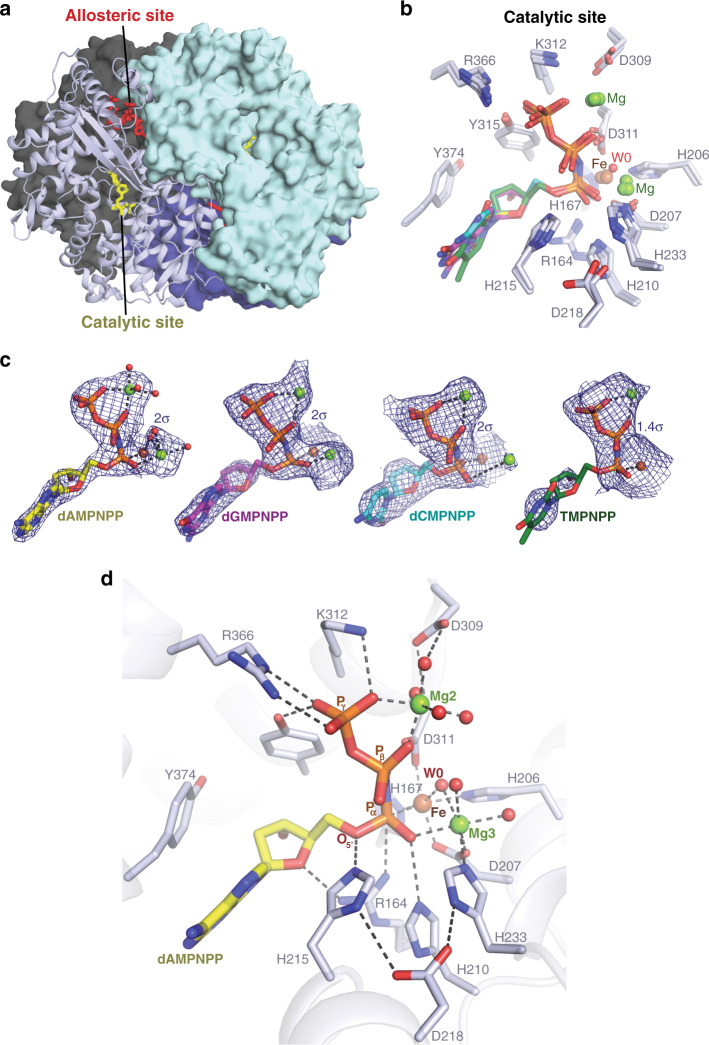
Crystal structures of SAMHD1–dNMPNPP inhibitor complexes. **a** Crystal structure of D137N-SAMHD1(109-626)-XTP-dAMPNPP inhibitor complex tetramer. Three monomers are shown in surface representation with a single monomer shown in cartoon representation. Monomers are coloured cyan, dark grey, dark blue and light blue, respectively. Nucleotides bound at the active and allosteric site in the cartoon monomer are shown in stick representation and coloured yellow and red, respectively. **b** Structural alignment of the active sites of the D137N-SAMHD1(109-626)-XTP-dAMPNPP, D137N-SAMHD1(109-626)-XTP-dGMPNPP, wt-SAMHD1(109-626)-GTP-dATP-dCMPNPP and wt-SAMHD1(109-626)-GTP-dATP-TMPNPP structures. Overlapping-bound nucleotides and active-site residues are shown in stick representation. The carbon atoms of the base and sugar of the bound nucleotides are coloured: yellow, dAMPNPP; magenta, dGMPNPP; cyan, dCMPNPP: and green, TMPNPP. **c** Simulated-annealing composite omit 2*F*_o_ − *F*_c_ electron density (blue mesh) for active site-bound nucleotides in D137N-XTP-dAMPNPP, D137N-XTP-dGMPNPP, wt-GTP-dATP-dCMPNPP and wt-GTP-dATP-TMPNPP structures. Nucleotides are shown in stick representation and colour coded as in **b**. Metal ions are shown as spheres (Mg, green; Fe, brown). **d** Detailed view of the active site of the D137N-SAMHD1(109-626)-XTP-dAMPNPP inhibitor complex. The protein backbone is shown in cartoon representation, bound nucleotides and active site residues are shown in stick representation, metal ions and ordered water molecules as spheres, coloured by atom type (oxygen, red; Mg, green; Fe, brown). Hydrogen bonding and metal ion coordinate bonds are shown as dashed lines. W0 indicates the in-line attacking nucleophilic water molecule.

The resolution of these structures varied between 2.0 and 3.2 Å, but all structures contain highly homologous “closed” tetramers of the SAMHD1 catalytic domain (Fig. 3a), with pairwise root-mean-square deviations (RMSDs) of <0.5 Å across all atoms in each tetramer. Moreover, although the D137N-XTP-dAMPNPP structure is resolved at the highest resolution, there is clear density for metal ions and nucleotides bound at the allosteric and active sites in all the structures, and the coordination of dNMPNPP nucleotides at the active sites is highly similar (Fig. 3b–d and Supplementary Fig. 5).

### Active-site configuration of SAMHD1–dNMPNPP complexes

Our SAMHD1–dNMPNPP structures share many similarities with the allosteric and active sites observed in previous structures^16,37,48,51^. However, with regards to the active-site nucleotide configuration and metal ion content, it is apparent there are significant differences that are crucial for understanding the catalytic mechanism of SAMHD1. This is most evident in our highest resolution structure, D137N-SAMHD1(109-626)-XTP-dAMPNPP-Mg, that clearly demonstrates how three octahedrally coordinated metal ions Fe, Mg2 and Mg3, support catalysis through interactions with the bound substrate, as detailed below.

Inspection of the D137N-XTP-dAMPNPP structure (Fig. 3d) reveals the key residues that position the α-phosphate proximal to the HD motif-coordinated iron, and are likely important for dNTP hydrolysis. In particular, the nucleotide 5′ oxygen, as well as non-bridging α-phosphate oxygens of dAMPNPP make hydrogen bonds with SAMHD1 side chains Arg164, His210 and His215, whereas the β- and γ-phosphates are coordinated by Mg2 (Fig. 3d). The two non-bridging α-phosphate oxygens form coordinate bonds with Fe and Mg3 (Fig. 3b–d), one phosphate oxygen coordinates Fe with an Fe–O bond distance of 2.2 Å and the second phosphate oxygen coordinates Mg3, with an Mg–O bond distance of 2.3 Å. Notably, the Fe and Mg3 metal ions have an interatomic spacing of 3.9 ± 0.5 Å (mean ± standard deviation across all Mg^2+^-SAMHD1–dNMPNPP structures) and are bridged by the carboxyl side chain of Asp207 from the HD motif as well as coordinated by the non-bridging α-phosphate oxygens of the dAMPNPP nucleotide and a water molecule, W0 (Fig. 3d).

These data reveal that the catalytic site of SAMHD1 actually comprises a bi-metallic Fe–Mg centre and although this has never been reported in previous SAMHD1-nucleotide structures, similar bi-metallic centres are present in other HD domains and several proteins that catalyse phosphoryl transfer reactions^59–63^. Therefore, given our observations, we sought to validate our findings and confirm the position of metal ions by substitution of Mg^2+^ with Mn^2+^ and identify ion positions using diffraction methods.

Although Mg^2+^ is essential for SAMHD1 catalysis, Mn^2+^ still supports dNTP hydrolysis with similar catalytic parameters (Supplementary Fig. 6a, b), as is observed in other phosphohydrolase enzymes. Therefore, Mg^2+^ ions were substituted with Mn^2+^ ions in the SAMHD1–dNMPNPP co-crystallisation conditions and the structure of D137N-SAMHD1(109-626)-Mn-XTP-dAMPNPP complex was determined at 2.25 Å (Supplementary Tables 3 and 4). 2*F*_o_ − *F*_c_ maps contoured at high *σ* were then used to locate and confirm the position of the highly X-ray scattering Mn^2+^ ions in the active and allosteric sites in the structure (Supplementary Fig. 6c, d). In addition, anomalous difference maps calculated from datasets recorded at 0.93 Å from a D137N-SAMHD1(109-626)-Mg-XTP-dAMPNPP complex and at the Mn and Fe absorbance edges on a D137N-SAMHD1(109-626)-Mn–Mg-XTP-dATP-dCMPNPP complex (Supplementary Fig. 7) were further used to locate Mn ions and confirm the presence of Fe bound at the HD motif, as observed previously^16^. Taken together, these data demonstrate that there are three manganese ions coordinated per SAMHD1 monomer, one occupying the magnesium-binding site at the allosteric site and a further two in the active site, at the Mg2 and Mg3 positions, with Fe and Mn3 forming a bi-metallic centre analogous to the Fe–Mg3 centre that we observe in our Mg^2+^-SAMHD1–dNMPNPP structures.

### The Fe–Mg centre positions a hydroxide nucleophile

**Fig. 4:**
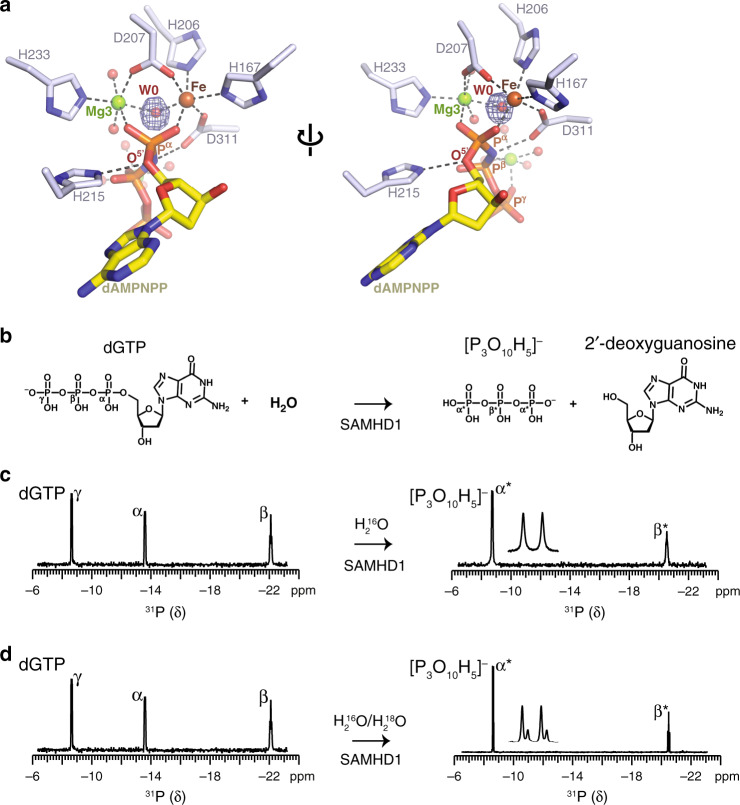
NMR analysis of water-mediated SAMHD1 catalysis. **a** Detailed view of the bi-metallic centre in the SAMHD1-active site. Metal ions and coordinated water molecules are shown as spheres, coloured by atom type. W0 is the activated water molecule positioned between the HD-bound Fe and Mg3, and orientated for nucleophilic attack on the α-phosphate. The electron density shown in blue mesh is the *F*_o_ − *F*_c_ map calculated during model building prior to inclusion of W0, contoured at 6 σ. **b** Chemical structures of the dGTP substrate and deoxyguanosine and triphosphate products of SAMHD1 hydrolysis, the α, β and γ-phosphate of dGTP together with the α*- and β*-phosphate of triphosphate are labelled. **c**, **d** SAMHD1 hydrolysis of dGTP monitored by ^31^P NMR. Spectra of dGTP substrate (left) and the triphosphate product (right) after 0 and 3 h incubation with SAMHD1 are shown. Assigned ^31^P resonances of the α-, β- and γ-phosphate of dGTP and α- and β-phosphate of the triphosphate product species are labelled. Product resonances are marked with asterisks. An expanded view of the α*-phosphate doublet resonance is shown inset. In **c** the reaction was carried out in solvent comprising 1:1 mixture of H_2_O^16^ and H_2_O^18^. The satellite peaks on α*-phosphate doublet resonance result from the O^18^ isotope effect, demonstrating that the reaction proceeds via a nucleophilic attack from a water molecule on the α-phosphate that is subsequently transferred into the triphosphate product.

Detailed inspection of our Mg^2+^-SAMHD1-dAMPNPP crystal structure revealed that, at the Fe–Mg centre, a water species, that we term W0, bridges the two metal ions with an approximately tetrahedral Fe–O–Mg bond angle (119 ± 11°; mean ± standard deviation) and that W0 is also in-line with the dAMPNPP P^α^-O^5′^ phosphoester bond (W0-P^α^-O^5′^ angle, 176.3 ± 0.7°; mean ± standard deviation) (Fig. 4a). Therefore, given this proximity and configuration, we hypothesised that W0 is the source of the nucleophile that attacks the P^α^ during SAMHD1-catalysed dNTP hydrolysis.

Support for this notion comes from studies showing that iron and magnesium reduce the p*K*_a_ for deprotonation of coordinated water molecules to form the more nucleophilic hydroxide species^64,65^. In addition, quantum chemical calculations show that the bridging water at the Zn–Mg or Mn–Mg bi-metallic centres in the HD domains of human 3′,5′-cyclic nucleotide phosphodiesterase (PDE) enzymes can partially dissociate at physiological pH to form a hydroxide ion^66–68^. Given that the SAMHD1 Fe–Mg and PDE bi-metallic centres, used in the quantum chemical calculations, are similarly coordinated (Fig. 3d and Supplementary Fig. 8), this adds further support to the idea that, in SAMHD1, the W0 Fe–Mg-bridging water also likely dissociates to form a hydroxide ion. This hydroxide ion then acts as the attacking nucleophile at the P^α^ on a substrate dNTP, resulting in cleavage of the P^α^-O^5′^ phosphoester bond.

To test this hypothesis with regard to the involvement of water in the SAMHD1 catalytic mechanism, the hydrolysis reaction of dGTP was monitored using ^31^P NMR spectroscopy (Fig. 4b–d). These solution NMR experiments clearly show the conversion of the substrate dGTP spectrum, containing the three resonances from phosphorus at positions α, β and γ, to the two peaks, α* and β*, from the triphosphate product (Fig. 4c).

In addition, to facilitate tracking of solvent water molecules, the hydrolysis reaction was conducted in the presence of a 1:1 mixture of H_2_^16^O and H_2_^18^O solvent. Here, the product α*-phosphorus resonance now has a satellite peak, resulting from the isotope shift upon covalent bonding of ^18^O, rather than ^16^O, to the α*-phosphorus, with a parent-to-satellite peak intensity ratio of ~3:1 (Fig. 4d). Therefore, these data show that the reaction proceeds through nucleophilic attack of a water molecule on the α-phosphorus, resulting in oxygen from bulk water being incorporated into the triphosphate product. Moreover, as the triphosphate product contains two equivalent α*-phosphorous atoms, the ~3:1 ratio of parent to satellite peak intensities of the product α*-phosphate reveals a stoichiometry of a single oxygen incorporation through initial adduction at the substrate dGTP α-phosphorus. Taken together, these observations support the notion of a Fe–Mg bi-metallic centre in the SAMHD1 catalytic site that positions a hydroxide ion for nucleophilic attack on a substrate dNTP α-phosphorus, which results in dissolution of the P^α^-O^5′^ phosphoester bond to release the triphosphate and 2′-deoxynucleoside products.

### Mechanism of SAMHD1-dNTP hydrolysis

**Fig. 5:**
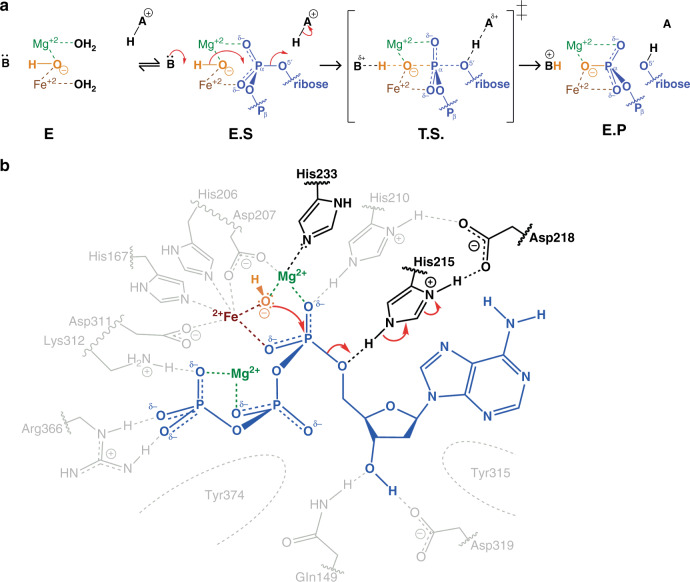
Catalytic mechanism of SAMHD1-dNTP hydrolysis. **a** Schematic of the chemical mechanism of SAMHD1-dNTP hydrolysis. In the apo state (E) the W0 water molecule (orange) is coordinated between the HD motif-bound Fe ion and by Mg3, further water molecules take up the remaining coordination positions on the metal ions. On substrate binding, the enzyme–substrate complex (E.S) is formed and P^α^ oxygens replace water molecules to coordinate the Mg3 and Fe ions and also position the W0 nucleophile in line with the electron deficient α-phosphate. The reaction proceeds by adduction of the W0 nucleophile to the α-phosphate to form a trigonal-bipyramidal intermediate transition state (T.S.). The resulting accumulating negative charge is relieved by protonation of the leaving nucleoside 5′ oxygen to form the enzyme product complex (E.P). **b** Schematic of SAMHD1-active site conformation during the hydrolysis reaction. A bound substrate dATP nucleotide is shown in blue, W0 in orange, the HD Fe ion in brown and active site Mg ions in green. The side chains of His233 and His215 and Asp218 required for catalysis are highlighted and the direction of electron transfer is indicated by the red arrows.

We propose that the crystal structures of our SAMHD1–dNMPNPP enzyme-inhibitor (EI) complexes represent good approximations of SAMHD1-dNTP enzyme–substrate (ES) complexes. Given these structural observations and our enzymological and NMR data, we propose the mechanism for SAMHD1-catalysed hydrolysis of dNTPs presented in Fig. 5a. In the mechanism, in the absence of a substrate dNTP, both Fe and Mg3 adopt distorted octahedral coordination with ligands provided by the side chains of (i) His167, Asp311, His206, Asp207, W0 and an additional water molecule for Fe; and (ii) Asp207, W0, His233 and three additional water molecules for Mg3. This is supported by the crystal structures of unliganded SAMHD1 that have either water or negatively charged phosphate bound at the active site Fe^4,52^.

In this configuration, W0 bridges Fe and Mg3 of the bi-metallic centre. This facilitates its partial dissociation to hydroxide at neutral pH to generate the nucleophile required for the hydrolysis reaction, an idea supported by previous quantum chemical calculations^66–68^. Upon binding of a substrate dNTP and formation of the ES complex, the two non-bridging P^α^ oxygens of the dNTP displace two of the water molecule ligands of Fe1 and Mg3 while maintaining the W0 position, the Fe–Mg3 spacing of 3.9 Å and the octahedral coordination geometry of the bi-metallic centre. In this configuration, the activated W0 hydroxide is now 2.8 Å away from the electron deficient P^α^, and the W0-P^α^-O^5′^ angle is 176°, strongly suggesting that a W0 hydroxide is the nucleophile of the hydrolysis reaction. The reaction then proceeds by nucleophilic attack by the W0 hydroxide at P^α^, and adduction to a form a trigonal-bipyramidal transition state (Fig. 5a, transition state (TS)). Inversion of P^α^ and breakage of the P^α^-O^5′^ bond results in incorporation of W0 into the newly formed triphosphate product and concomitant release of the 2′-deoxynucleoside.

A requirement of the mechanism is that during the ES to EP transition, a general acid is required for protonation of the O^5′^ in order for the P^α^-O^5′^ phosphoester bond to break and allow leaving of the deoxynucleoside product. In our SAMHD1–dNMPNPP crystal structures, the Nε2 of the conserved His215 side chain makes a hydrogen bond with the O^5′^ and is ideally placed to stabilise evolving negative charge during the transition from ES to EP and also donate a proton to the leaving 2′-deoxynucleoside (Fig. 5b). In addition, the side chain Nδ1 proton of His215 is shared with the acidic side chain of the conserved Asp218, and this configuration likely contributes further to charge neutralisation through proton relay. Therefore, the hydrolytic mechanism we propose is consistent with our experimental data demonstrating the requirement of Mg^2+^ for catalysis, the presence of a bi-metallic centre and hydroxide nucleophile in the SAMHD1 catalytic site and the proximity of highly conserved catalytic histidine residues, His215 and His233, to the metal centre and the labile P^α^-O^5′^ bond.

### Critical catalytic residues

**Fig. 6:**
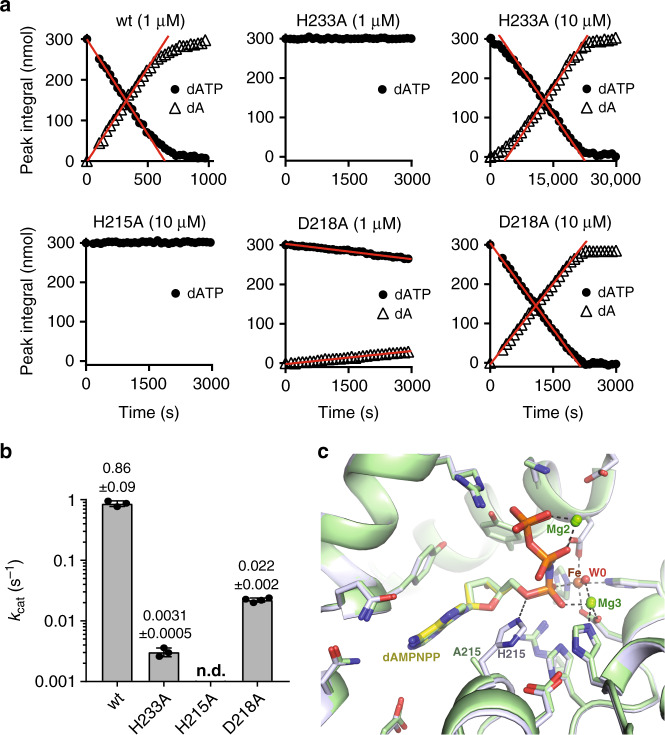
Catalytic activity of SAMHD1-active site mutants. **a** ^1^H NMR analysis of GTP-stimulated dATP hydrolysis by wt-SAMHD1 and active site mutants H233A, H215A and D218A. ^1^H NMR data were recorded for SAMHD1 hydrolysis reactions containing wt-SAMHD1 (1 µM) or active site mutants H233A (1 and 10 µM), H215A (10 µM) and D218A (1 and 10 µM) with 0.2 mM GTP AL1-activator and 0.5 mM dATP. In each panel, the integral of resolved substrate and product peak resonances is plotted against time. Initial rates of hydrolysis were determined from slopes (red lines) derived from the data measured in the linear part of the reaction. **b** Bar chart of the apparent *k*_cat_ values derived from the data shown in **a**. Points overlaid in each bar are the individual data measurements, the error bars represent the s.d. of at least three independent measurements, n.d. (not detectable). The source data are provided in the Source Data file. **c** Overlay of the active site in the crystal structures of D137N-SAMHD1(109-626)-XTP-dApNHpp and H215A–SAMHD1(109-626)-GTP-dAMPNPP inhibitor complexes. The protein backbone is shown in cartoon representation, wt (grey) and H215A (green), bound nucleotides and active site residues are shown in stick representation, metal ions and ordered water molecules as spheres, coloured by atom type. The bound inhibitor adopts the same configuration in both the wt and H215A structures.

In order to validate our reaction mechanism, site-directed mutagenesis and enzymological studies were employed to probe the function of active site histidine residues required for different aspects of catalysis. First, to test the importance of magnesium coordination at the Fe–Mg3 centre, His233, which makes a coordinate bond with Mg3 (Figs. 4a and 5b), was mutated to alanine to perturb magnesium binding. The introduction of the H233A substitution still supported tetramerisation upon addition of GTP and dATP or dAMPNPP at levels similar to wt-SAMHD1 as observed by SEC-MALLS (Supplementary Fig. 9a–d) indicating that formation of the active tetrameric state is not compromised. However, in agreement with previous observations^52^, the apparent *k*_cat_ for GTP-activated dATP hydrolysis was reduced ~300-fold, as determined using ^1^H NMR spectroscopy (Fig. 6a, b). Therefore, these data are consistent with our model where magnesium coordination by His233 at the SAMHD1 Fe–Mg bi-metallic centre is important for dNTP coordination and hydrolysis.

In our proposed mechanism, His215 is the general acid that stabilises evolving negative charge at the O^5′^ during the ES to EP transition and likely protonates the O^5′^ on the 2′-deoxynucleoside leaving group (Fig. 5b). Asp218 is another highly conserved residue that makes a charged hydrogen bond to the side chain of His215, stabilising a positive charge on the Nδ1 of His215 and making the Nε2 proton available and proximal to the O^5′^ (Fig. 5b). Therefore, to probe the contribution of His215 and Asp218 to the hydrolysis reaction, alanine substitution mutants, H215A and D218A were also assessed for tetramerisation and triphosphohydrolase activity using SEC-MALLS and ^1^H NMR spectroscopy, respectively. Both mutants, H215A and D218A, supported tetramerisation upon addition of GTP and dATP or dAMPNPP at levels similar to wt-SAMHD1 (Supplementary Fig. 9e–h) and so can form the active tetrameric state. However, no measurable triphosphohydrolase activity could be detected for the H215A mutant (Fig. 6a, b), even when elevated levels (10 µM) of H215A–SAMHD1 were employed in the assay, confirming its critical function in catalysis. Moreover, the D218A substitution, although not entirely abolishing activity, heavily reduced the apparent *k*_cat_ by ~40-fold (Fig. 6a, b), consistent with its stabilising function in catalysis through maintenance of the His215 protonation state and orientation of the side chain close to the P^α^-O^5′^ phosphoester bond.

To further test the importance of His215 in the catalytic mechanism, we also determined the crystal structure of a mutant H215A–SAMHD1(109-626)-GTP-dAMPNPP inhibitor complex, details of the structure refinement and unit cell contents are presented in Supplementary Tables 3 and 4. In the structure, the bound active site dAMPNPP nucleotide adopts the same configuration as that observed in the wt and D137N structures. A 3D structural superimposition of the H215A and wt structures based on alignment of all SAMHD1 atoms reveals an overall rmsd of only 0.531 Å. In addition, the active site side chains, metal ions and water molecule positions are also maintained in the same positions as in the wt active site (Fig. 6c and Supplementary Fig. 10) further supporting His215 function in catalysis at a stage subsequent to tetramer formation and binding of the substrate.

## Discussion

SAMHD1 regulates the cellular dNTP pool through its dNTP triphosphohydrolase activity. However, despite the availability of several SAMHD1-nucleotide co-crystal structures, the structural basis for catalysis remained unclear. Therefore, we sought to determine: (i) how a substrate dNTP is coordinated at the catalytic site; and (ii) how SAMHD1 catalyses dNTP triphosphohydrolysis. Using combined biophysical and X-ray structural studies incorporating nucleotide analogues along with mutagenesis experiments we have now discovered that SAMHD1 uses a bi-metallic Fe–Mg centre to catalyse dNTP hydrolysis in a reaction similar to those catalysed by HD domains in both human and bacterial phosphodiesterase PDE enzymes^59–63^. Moreover, we have obtained a clear picture of substrate and inhibitor binding at the SAMHD1-active site enabling us to propose a detailed chemical mechanism for dNTP hydrolysis catalysed by SAMHD1.

We determined co-crystal structures of wt- or D137N-SAMHD1 HD catalytic domains with the dAMPNPP, dGMPNPP, dCMPNPP and TMPNPP non-hydrolysable dNTP analogues coordinated in the catalytic site (Fig. 3). Unlike previous high-resolution structures of the human SAMHD1 HD catalytic domain, that have largely employed metal ion disrupting H206R/D207N HD-mutants to catalytically inactivate SAMHD1^37,48,51^, in our structures the active site is entirely native. In addition, we solved a further structure of the catalytically inactive mutant H215A, which does not disrupt metal ion binding, with dAMPNPP bound in the active site. In all our structures the active site side chain configurations, nucleotide conformation and metal ion–water network are all conserved, including in the H215A structure (Fig. 6c). These observations support the notion that, although critical for catalysis, His215 is not required for substrate binding. In addition, our structures reveal that a bi-metallic Fe–Mg centre at the SAMHD1-active site positions a water moiety, W0, in-line with the scissile P^α^-O^5′^ bond of a substrate dNTP, providing a model for the SAMHD1 enzyme–substrate complex (ES) prior to formation of the transition state.

Several features of our ES complex model have been observed in previously reported SAMHD1 structures^37,48,49^. These include selectivity for 2′-deoxyribose through hydrogen bonding between the dNTP 3′-OH with Gln149 and Glu319, and steric hindrance from residues Leu150 and Tyr374 preventing binding of a ribose (2′R)-2′-OH NTP, as well as the stabilisation of dNTP binding at the catalytic site through hydrogen bonds and salt-bridges between dNTP phosphate oxygens and residues Arg164, His210, Lys312 and Arg366. However, while 2′-deoxyribose- and base-coordination in the structures presented here share these similarities with previous structures, it is apparent that the position of the dNTP α-phosphate together with water molecule organisation and metal ion complement and coordination is very different to that observed previously. Therefore, our observations, not only build significantly on previous studies, but given we observe the same configuration in different space groups and with different nucleotides and active site mutants, we are confident that our structures provide an accurate representation of the ES complex prior to substrate dNTP hydrolysis by SAMHD1.

Our enzymological data clearly demonstrate that, although the α,β-methyleno dNMPCPP analogues are SAMHD1 substrates, the α,β-imido dNMPNPP analogues in our structures are competitive inhibitors of SAMHD1, with nM *K*_i_ values, much lower than the apparent *K*_*M*_ values reported for canonical nucleotides that are in the range of 10^−5^ to 10^−3^ M^35,37,69^. Analysis of the dNMPNPP co-complex crystal structures now suggests why this is the case. In the active site of the dNMPNPP structures, regardless of the nucleotide base, it is apparent that the side chain of Asp311 of the HD motif not only makes a coordinate bond with the bound active site Fe but also acts as an acceptor in a hydrogen bonding interaction with the proton (H^imido^) of the α,β-imido group of the dNMPNPP (Figs. 3 and 6). Moreover, the active site Fe ion is highly polarising and likely distorts the delocalised carboxyl Asp311 side chain, to further increase the strength of this hydrogen bond. By contrast, canonical dNTPs and dNMPCPP analogues that are all substrates are unable to make an equivalent hydrogen bonding interaction with Asp311. Canonical nucleotides have a α,β-bridging oxygen at this position, which has a low p*K*_a_ and so have no proton to donate and the methyleno C–H group in the dNMPCPP analogues is too weakly polarised to donate a proton in a hydrogen bonding interaction with Asp311.

Given these observations, our preferred hypothesis for competitive inhibition of SAMHD1 by dNMPNPP nucleotides is that this hydrogen bond, between Asp311^Oδ^ and H^imido^ stabilises the EI complex to the extent that the reaction cannot proceed to the trigonal-bipyramidal transition state illustrated in Fig. 5a and thus prevents P^α^ inversion and P^α^-O^5′^ bond cleavage. In addition, a smaller contribution from electronegativity differences of the α,β-bridging O, N and C in dNTPs, dNMPNPPs and dNMPCPPs respectively, and the effect of this on the reactivity of the adjacent α-phosphate, may also contribute and explain our observation that canonical dNTPs are hydrolysed ~3-fold faster than dNMPCPPs, due to the greater electronegativity of oxygen over carbon increasing the reactivity of the adjacent P^α^ for nucleophilic attack. Nevertheless, the stabilising effect of the Asp311^Oδ^-H^imido^ hydrogen bond likely dominates and most importantly the dNMPNPP analogues are non-hydrolysable competitive inhibitors of SAMHD1 that bind tightly to the active site with low *K*_i_ constants. Therefore, our SAMHD1–dNMPNPP co-crystal structures provide an opportunity for the development of SAMHD1 inhibitors that can be employed in a cellular context.

Different metal ions have been reported to coordinate the HD motif residues His167, His206, Asp207 and Asp311 at the SAMHD1 catalytic site. In wt-SAMHD1-nucleotide complexes, iron, zinc and manganese have all been reported to coordinate the HD motif^4,16,48,49^. By contrast, in structures of the catalytically inactive double mutant H206R/D207N, the HD motif metal ion-binding site is unoccupied, and instead magnesium is coordinated by the dNTP β- and γ-phosphates, proximally to Asp309^48^. In our SAMHD1-nucleotide structures, both of these metal ion-binding sites in the catalytic site are occupied, with iron coordinated by the HD motif, and magnesium (Mg2) by the dNTP β- and γ-phosphates (Fig. 3). However, additionally in our structures it is apparent that a further magnesium ion, Mg3, is also coordinated at the catalytic site by Asp207 of the HD motif and by the proximally located His233 (Figs. 3d and 4a). Critically, Mg3 along with Fe form the Fe–Mg bi-metallic centre that is coordinated by the carboxyl side chain of Asp207, the non-bridging oxygens of the nucleotide α-phosphate and the water molecule, W0 (Fig. 4a). Given we postulate that the Fe–Mg3-bridging W0, is activated by its environment to form the hydroxide nucleophile for in-line attack on the dNTP P^α^-O^5′^ bond, the metal ion configuration at the SAMHD1-active site has important implications for understanding how SAMHD1 interacts with nucleotides and nucleotide analogues and how it catalyses hydrolysis.

Our SAMHD1 catalytic mechanism critically relies on a hydroxyl nucleophile that bridges an Fe–Mg bi-metallic centre. In other HD domains, bi-metallic centres have also been shown to be necessary for catalytic activity^59–63^. Structural studies of reaction intermediates of the HD containing human 3′,5′-cyclic nucleotide phosphodiesterase 9 (PDE9) clearly demonstrate a water-bridged bi-metallic centre in the active site, where the cGMP substrate is posed for nucleophilic attack at P^α^ by a Mn–Mg-bridging W0 (Supplementary Fig. 8)^59^. Here, the activated water molecule lies approximately in-line with the P^α^-O^3′^ bond that is cleaved in the cGMP hydrolysis reaction catalysed by PDE9 and is analogous to W0 in the mechanism that we postulate for SAMHD1 catalysis of P^α^-O^5′^ bond cleavage in dNTP substrates. Further, His215 and Asp218 in SAMHD1 and His252 and Glu423 in PDE9 appear to play equivalent roles in both mechanisms, acting as a proton relay to protonate the O^5′^ of the deoxynucleoside leaving group.

In bacteria, some HD domains have been exapted into signalling pathways where the bi-metallic centres of the 7TMR-HD and HD-GYP protein families^60,70^ catalyse phospho-hydrolysis of the chemical messengers cyclic-di-AMP and cyclic-di-GMP, respectively^60,62,63^. Elsewhere, in the mammalian enzyme myo-inositol oxygenase (MIOX)^71^ and the bacterial enzyme PhnZ^72,73^ HD domains have evolved to catalyse a different chemical reaction, oxidative carbon-halogen (C-X) bond cleavage. Therefore, it is clear that the bi-metallic centres of diverse HD domain-containing enzymes are used to catalyse a broad range of chemical reactions. However, despite variations in the reactions catalysed, they appear to share the use of a carboxyl side chain of an Asp or Glu residue to bridge the adjacent metal ions. Thus, although we find that the coordination about the Fe–Mg bi-metallic centre of SAMHD1 appears chemically unique, in fact SAMHD1 catalyses nucleotide hydrolysis in a similar manner to the hydrolysis reactions carried out by the HD domains of PDEs^59,61^ as well as 7TMR-HD^60^ and HD-GYP^62,63^ enzymes.

The wealth of data we have accumulated relating to SAMHD1 catalysis and catalytic mechanism now realises our understanding of how SAMHD1 catalyses dNTP hydrolysis to regulate the cellular dNTP pool. Moreover, the description of the competent configuration of SAMHD1 substrates and the ES complex informs on how to avoid the effects of SAMHD1 hydrolysis on new nucleotide based-therapies and goes some way to the development of inhibitors that might be used to modulate SAMHD1 activity in a cellular context to understand function in HIV-1 restriction, DNA repair and innate immune sensing.

## Methods

### Protein expression and purification

For expression in *E. coli*, the DNA sequences coding for human SAMHD1 residues M1-M626, SAMHD1 and Q109-M626, SAMHD1(109-626), were amplified by PCR and inserted into a pET52b expression vector (Novagen) using ligation independent cloning (SAMHD1) or the *Xma*1/*Not*1 restriction sites (SAMHD1(109-626)) to produce N-terminal StrepII-tag fusion proteins. The D137N and active site point mutants were prepared from the parent SAMHD1 and SAMHD1(109-M626) constructs using the Quikchange II kit. Primer sequences for PCR and mutagenesis are provide in Supplementary Table 5 and all insert sequences were verified by DNA sequencing. StrepII-tagged SAMHD1 constructs were expressed in the *E. coli* strain Rosetta 2 (DE3) (Novagen) grown at 37 °C with shaking. Protein expression was induced by addition of 0.1 mM IPTG to log phase cultures (*A*_600_ = 0.5) and the cells incubated for a further 20 h at 18 °C. Cells were harvested by centrifugation, resuspended in 50 mL lysis buffer; 50 mM Tris-HCl pH 7.8, 500 mM NaCl, 4 mM MgCl_2_, 0.5 mM TCEP, 1× EDTA-free mini complete protease inhibitors (Roche) per 10 g of cell pellet and lysed by sonication. The lysate was cleared by centrifugation for 1 h at 50,000×*g* and 4 °C, and subsequently applied to a 10 mL StrepTactin affinity column (IBA), followed by 300 mL of wash buffer (50 mM Tris-HCl pH 7.8, 500 mM NaCl, 4 mM MgCl_2_, 0.5 mM TCEP) at 4 °C. Bound proteins were eluted from the column by circulation of 0.5 mg of PreScission Protease (GE) in 25 mL of wash buffer over the column in a closed circuit overnight. The supernatant (25 mL) and an additional elution of wash buffer (15 mL) were pooled and concentrated to 2.5 mL. PreScission Protease was removed by affinity chromatography using a 1 mL GSTrap HP column (GE) and the eluent was applied to a Superdex 200 26/60 (GE) size exclusion column equilibrated with a gel filtration buffer of 10 mM Tris-HCl pH 7.8, 150 mM NaCl, 4 mM MgCl_2_, 0.5 mM TCEP. Peak fractions were concentrated to approximately 20 mg mL^−1^ and flash-frozen in liquid nitrogen in small aliquots.

### Nucleotides

GTP and dNTPs were purchased from ThermoFisher Scientific. XTP, and dNMPNPP and dNMPCPP dNTP analogues were purchased from Jena Bioscience, DE.

### Crystallisation and structure determination

We determined the X-ray crystal structures of the following seven complexes of SAMHD1(109-626), with canonical nucleotides and dNMPNPP analogues: (i) D137N-SAMHD1(109-626)-Mg-XTP-dAMPNPP, (ii) D137N-SAMHD1(109-626)-Mg-XTP-dGMPNPP, (iii) wt*-*SAMHD1(109-626)-Mg-GTP-dATP-dCMPNPP, (iv) wt*-*SAMHD1(109-626)-Mg-GTP-dATP-TMPNPP, (v) D137N-SAMHD1(109-626)-Mn-XTP-dAMPNPP, (vi) D137N-SAMHD1(109-626)-Mn–Mg-XTP-dATP-dCMPNPP and (vii) H215A–SAMHD1(109-626)-Mg-GTP-dAMPNPP. Prior to crystallisation, protein samples were diluted to 5 mg mL^−^^1^ with gel filtration buffer, and incubated with the appropriate nucleotides, as detailed above, at the following concentrations: XTP (1 mM), GTP (1 mM), dATP (0.5 mM) and dNMPNPP analogues (2 mM). For complexes v and vi, MnCl_2_ was also included at 5 mM.

Crystals were produced by sitting drop vapour diffusion at 18 °C using a mosquito® crystal robot (SPT Labtech) to prepare 0.2 µL droplets containing an equal volume of the protein/nucleotide solution and mother liquor. Crystals were obtained using a mother liquor of 100 mM Bis-tris-HCl pH 6–7.3 and 15–19% (v/v) PEG 3350, which additionally contained 150 mM Li_2_SO_4_ for complexes (ii), (iii) and (iv), and 100 mM MgCl_2_ for complex (vii). For data collection, 30% (v/v) glycerol was added to the respective crystallisation condition and crystals flash-frozen in liquid nitrogen. Datasets for determining the structures were collected on beamlines i03, i04, i04-1 and i24 at Diamond Light Source, UK, at wavelengths between 0.9282 and 0.9795 Å, with additional datasets for complex (vi) recorded at 1.9075 Å and at the manganese and iron *K* absorption edges, 1.8929 and 1.704 Å respectively. Details of the data collection along with crystallographic space groups, contents of the asymmetric unit and structure refinement statistics, are in Supplementary Tables 3 and 4.

Data were processed using either the autoPROC pipeline^74^ (Global Phasing LtD) for structures i to iv, or the DIALS pipeline^75^ for structures v, vi and vii. Internally, indexing and integration utilised XDS^76^ or DIALS; point-group symmetry was determined with POINTLESS^77^, isotropic scaling was carried out using AIMLESS^78^ and structure factors generated using CTRUNCATE^79^. For structures i-iv, the data were also anisotropically scaled in autoPROC using STARANISO (http://staraniso.globalphasing.org/cgi-bin/staraniso.cgi) (Global Phasing LtD).

Structures were solved by molecular replacement using the programme PHASER^80^ implemented in the CCP4 interface^81^ using the structure of wt-SAMHD1(113-626) (PDB code: 4BZC)^48^ as an initial search model to solve the D137N-SAMHD1(109-626)-Mg-XTP-dAMPNPP structure. Here, Buccaneer^82^ and manual building within the programme Coot^83^ were combined iteratively with refinement utilising individual B-factors and TLS groups in Refmac5^84^ to produce a final model for residues 114–275 and 284–599 (chain A), and residues 114–276 and 284–599 (chain B). The protein model produced for D137N-SAMHD1(109-626) in structure i was then used as the search model for molecular replacement with PHASER in datasets ii, v, vi and vii and the protein model produced in structure ii was used as the search model for molecular replacement with PHASER in datasets iii–iv. For refinement in Refmac5, the same common set of Free-R flags was applied to reflections shared between datasets i, v and vi, and a different common set of Free-R flags for reflections in datasets ii–iv. Simulated-annealing composite omit and anomalous difference maps were generated using phenix.maps within the Phenix software package^85^. The programme AceDRG^86^ was used to derive stereochemical restraint libraries for the nucleotide analogues: XTP, dAMPNPP, dGMPNPP, dCMPNPP and TMPNPP.

The coordinates and structure factors of the D137N-SAMHD1(109-626)-Mg-XTP-dAMPNPP, D137N-SAMHD1(109-626)-Mg-XTP-dGMPNPP, wt*-*SAMHD1(109-626)-Mg-GTP-dATP-dCMPNPP, wt*-*SAMHD1(109-626)-GTP-dATP-TMPNP, D137N-SAMHD1(109-626)-Mn-XTP-dAMPNPP, D137N-SAMHD1(109-626)-Mn-XTP-dATP-dCMPNPP and H215A–SAMHD1(109-626)-Mg-GTP-dAMPNPP inhibitor complexes have been deposited in the Protein Data Bank under accession numbers 6TX0, 6TXA, 6TXC, 6TXE, 6TXF, 6YOM and 6XU1, respectively.

### SEC-MALLS

Size exclusion chromatography coupled to Multi-Angle Laser Light Scattering (SEC-MALLS) was used to determine the molar mass composition of SAMHD1 samples upon addition of nucleotide analogues and activators. 30 µM wt-SAMHD1(1-626) was incubated at room temperature for 5 min after the addition of nucleotide analogues (0.5 mM) and activators (0.2 mM GTP). Samples (100 µL) were then applied to a Superdex 200 10/300 INCREASE GL column equilibrated in 20 mM Tris-HCl pH 7.8, 150 mM NaCl, 5 mM MgCl_2_, 0.5 mM TCEP and 3 mM NaN_3_ at a flow rate of 1.0 mL min^−1^. The scattered light intensity and protein concentration of the column eluate were recorded using a DAWN-HELEOS laser photometer and an OPTILAB-rEX differential refractometer (dRI) (d*n*/d*c* = 0.186), respectively. The weight-averaged molecular mass of material contained in chromatographic peaks was determined using the combined data from both detectors in the ASTRA software version 6.1 (Wyatt Technology Corp., Santa Barbara, CA).

### NMR analysis of SAMHD1 catalysis

1D ^1^H NMR spectroscopy was used to measure SAMHD1 hydrolysis rates of dNTPs. Reactions were prepared in NMR buffer (20 mM Tris-HCl pH 8.0, 150 mM NaCl, 5 mM MgCl_2_, 2 mM TCEP, 5% D_2_O) containing 0.5 mM of each dNTP or dNTP analogue, 0.2 mM GTP and 1–10 μM of wt- or mutant SAMHD1(1-626). In experiments designed to enhance hydrolysis of dNMPCPP analogues, 10 µM dATP was also included in the reaction. ^1^H NMR spectra (2 dummy scans, 4 scans) were recorded at 30 s intervals as a pseudo 2D array at 293 K using either a Bruker Avance III 600 MHz or Avance IIIHD 700 MHz NMR spectrometer equipped with a 5 mm TCI cryoprobe. Solvent suppression was achieved using excitation sculpting^87^. Experiments were typically carried out for between 1 and 10 h. The integrals for clearly-resolved substrate and product peaks at each time-point were extracted using the Bruker Dynamics Centre software package, and used to construct plots of substrate or product against time. Initial rates were extracted from the linear part of the curve in order to determine apparent *k*_cat_ values.

For ^31^P experiments, NMR Spectra were recorded at 25 °C on a 600 MHz Varian Inova spectrometer using a broadband X-nucleus detection probe. ^1^H decoupling, when employed, was achieved using the WALTZ scheme (B_1_ ~1600 Hz)^88^. SAMHD1 hydrolysis of 1 mM dGTP was monitored in a reaction buffer containing 1 µM wt-SAMHD1(1-626) in 20 mM Tris pH 8.0, 150 mM NaCl, 5 mM MgCl_2_ and 2 mM TCEP by recording a continuous series of 15-minute 1D spectra over a 6-hour period. Spectra were referenced indirectly to the ^1^H peak of the water using *γ*(^31^P)/*γ*(^1^H) = 0.404808636^89^. To assess the contribution of activated water to nucleophilic addition in the triphosphohydrolase reaction, a hydrolysis reaction was also prepared in standard reaction buffer but with a solvent comprising an equimolar mixture of H_2_^16^O and H_2_^18^O water. Hydrolysis of dGTP was measured using the same data acquisition parameters that were employed in the H_2_^16^O reaction.

### Real-time measurement of triphosphohydrolase activity

To obtain quantitative kinetic parameters for substrate hydrolysis (*K*_M_ and *k*_cat_) and for inhibition by dNMPNPP analogues (*K*_i_) a coupled assay was employed utilising the biosensor MDCC-PBP^90,91^ to measure phosphate release from combined SAMHD1 triphosphohydrolase and *S. cerevisiae* Ppx1 exopolyphosphatase activity^35^. In a typical experiment, solutions containing wt-SAMHD1(1-626), Ppx1, MDCC-PBP and GTP were incubated for 5 min in assay buffer (20 mM Tris pH 8.0, 150 mM NaCl, 5 mM MgCl_2_ and 2 mM TCEP) at 25 °C before the reaction was initiated by the addition of nucleotides and nucleotide analogues. The final concentrations were 0.2 µM SAMHD1, 0.01 µM Ppx1, 40 µM MDCC-PBP, 0.2 mM GTP and varying concentrations of dNTP substrates and analogues. The fluorescence intensity was recorded at 430 nm excitation and 465 nm emission over a period of 10–30 min in a Clariostar multiwell plate reader (BMG Labtech). Steady-state rates were obtained from time courses of *P*_i_ formation by linear regression of the data points in the linear phase of the reaction. Rates were divided by the SAMHD1 concentration and plotted versus substrate concentration. Apparent dissociation constants for substrate binding (*K*_M_) and catalytic constants (*k*_cat_) were then determined by non-linear least-squares fitting using a hyperbolic function in the software package Prism 7 (Graphpad).

For inhibition studies, experiments were conducted at three constant substrate concentrations (1, 0.3 and 0.1 mM TTP), the SAMHD1, Ppx1, MDCC-PBP and GTP concentrations were maintained as above and the inhibitor concentration was varied. Data from the three different experiments were analysed by non-linear least-squares fitting using the equation for competitive inhibition; where *V*/[SAMHD1] is the steady-state rate, normalised to the SAMHD1 concentration, [*S*] is the (fixed) substrate concentration, [*I*] is the (variable) inhibitor concentration, *K*_*i*_ is the inhibition constant, and *k*_cat_ and *K*_M_ are the catalytic and Michaelis–Menten constants for substrate turnover in the absence of inhibitor.

1 $$\frac{V}{{[{\mathrm{SAMHD1}}]}} = \frac{{k_{{\mathrm{cat}}} \cdot [S]}}{{[S] + K_{\mathrm{M}} \cdot \left( {1 + \frac{{[I]}}{{K_{\mathrm{i}}}}} \right)}}.$$

The fitting was performed with a fixed value of *K*_M_ for GTP-activated TTP hydrolysis, determined previously^35^ and only *k*_cat_ and *K*_i_ were allowed to vary. The fits for the three datasets yielded invariant values for *K*_i_ and *k*_cat_ supporting a competitive mode of inhibition. Global fitting of the entire three concentration dataset was used to extract the final *K*_i_ value. All measurements were performed in at least triplicate.

### Reporting summary

Further information on experimental design is available in the Nature Research Reporting Summary linked to this paper.

## Supplementary information

Supplementary Information

Reporting Summary

## Data Availability

The structural data that support the findings of this study have been deposited in the Protein Data Bank. The coordinates for the D137N-SAMHD1, Mg, XTP, dAMPNPP; D137N-SAMHD1, Mg, XTP, dGMPNPP; wt-SAMHD1, Mg, GTP, dATP, dCMPNPP; wt-SAMHD1, Mg, GTP, dATP, dTMPNPP; D137N-SAMHD1, Mn, XTP, dAMPNPP; D137N-SAMHD1, Mn, Mg, XTP, dATP, dCMPNPP; H215A–SAMHD1, Mg, GTP, dAMPNPP inhibitor complexes have PDB accession numbers 6TX0, 6TXA, 6TXC, 6TXE, 6TXF, 6YOM and 6XU1, respectively. Source data underlying Figs. 1b, c, 2b, 6a, b and Supplementary Figs. 4b, c and 6a, b are provided as a Source Data file. Other data that support the studies’ findings are available from the corresponding author upon reasonable request.

## Source data

Source Data
