# Supplementary material for: Crystal structures of SAMHD1 inhibitor complexes reveal the mechanism of water-mediated dNTP hydrolysis
Source: Nat Commun. 2020 Jun 23;11:3165. doi: 10.1038/s41467-020-16983-2 (PMC7311409; doi:10.1038/s41467-020-16983-2)
Supplement: Supplementary file 1 — Supplementary Information [file 41467_2020_16983_MOESM1_ESM.pdf]

Supplementary information

Crystal structures of SAMHD1 inhibitor complexes reveal the mechanism of water-mediated dNTP hydrolysis

Elizabeth R. Morris et al.

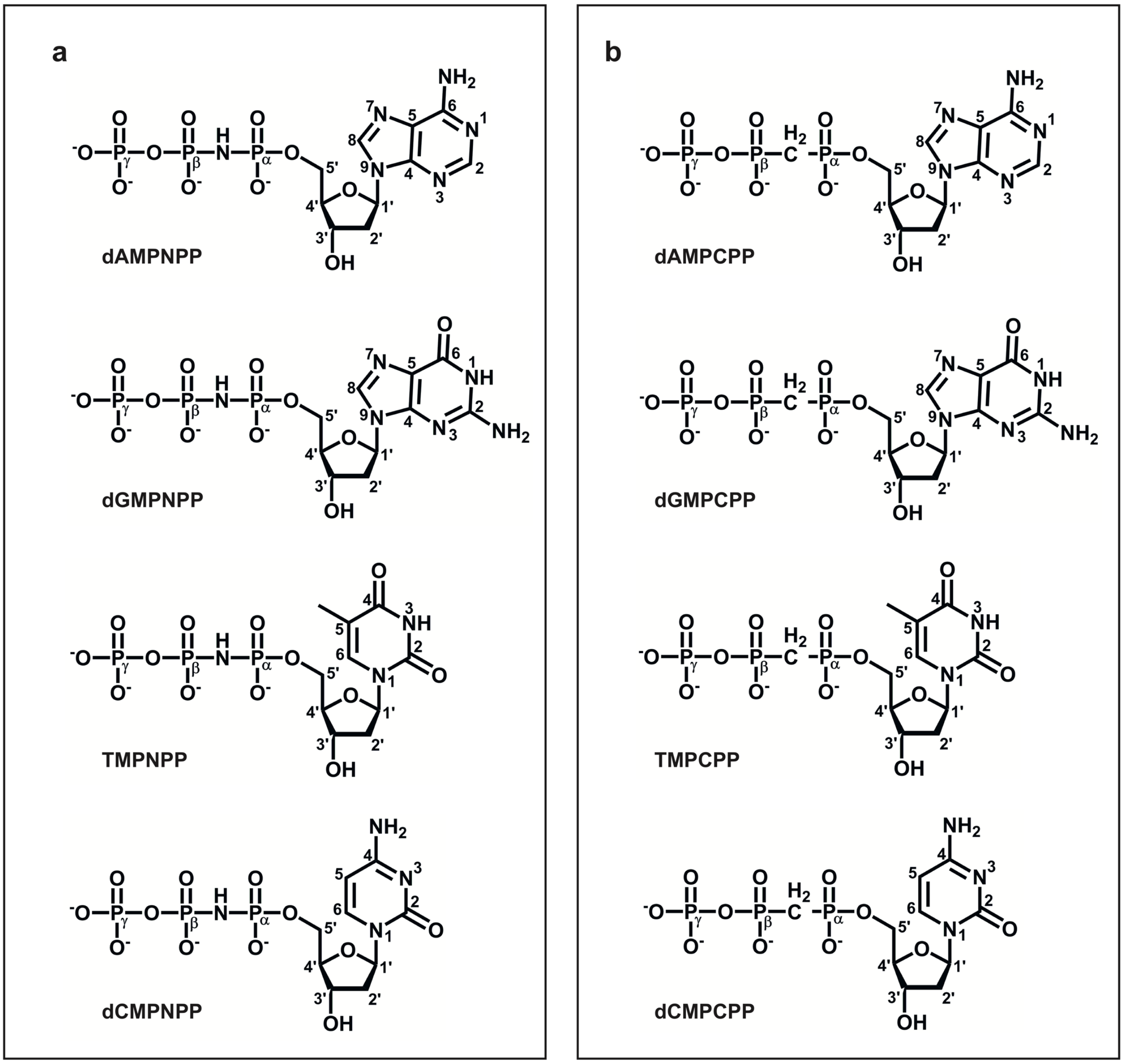

Supplementary Fig. 1. Chemical structures of deoxynucleotide analogues. Diagrammatic representations of the chemical structures of the (**a**) α,β imido (dNMPNPP) and (**b**) α,β methyleno (dNMPCPP) dNTP analogues employed in this study. Base and sugar carbon and nitrogen atoms are numbered using the standard convention for purine- and pyrimidine-based nucleotides.

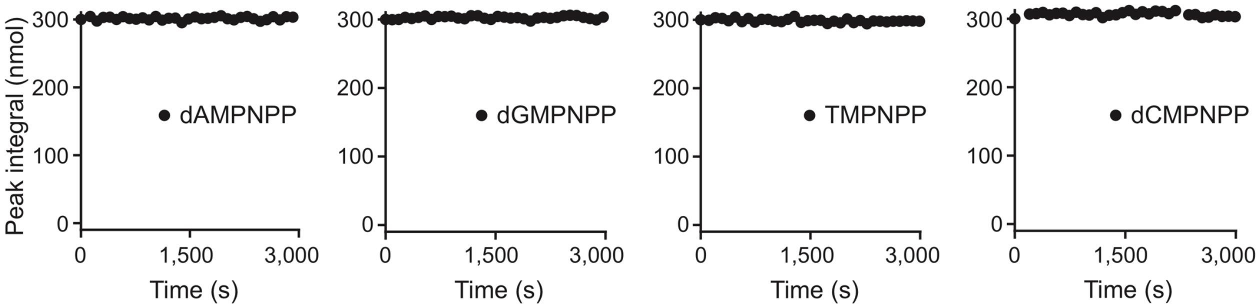

Supplementary Fig. 2. dNMPNPP analogues are not hydrolysed by SAMHD1. ^{1}H NMR Data recorded from a SAMHD1 hydrolysis reaction containing 1 µM SAMHD1, 0.2 mM GTP activator and 0.5 mM of dAMPNPP, dGMPNPP, TMPNPP or dCMPNPP. In each panel, the integrated area of resolved resonances from each dNMPNPP analogue is plotted against time. No significant reduction in peak intensities is apparent, indicating there is no hydrolysis.

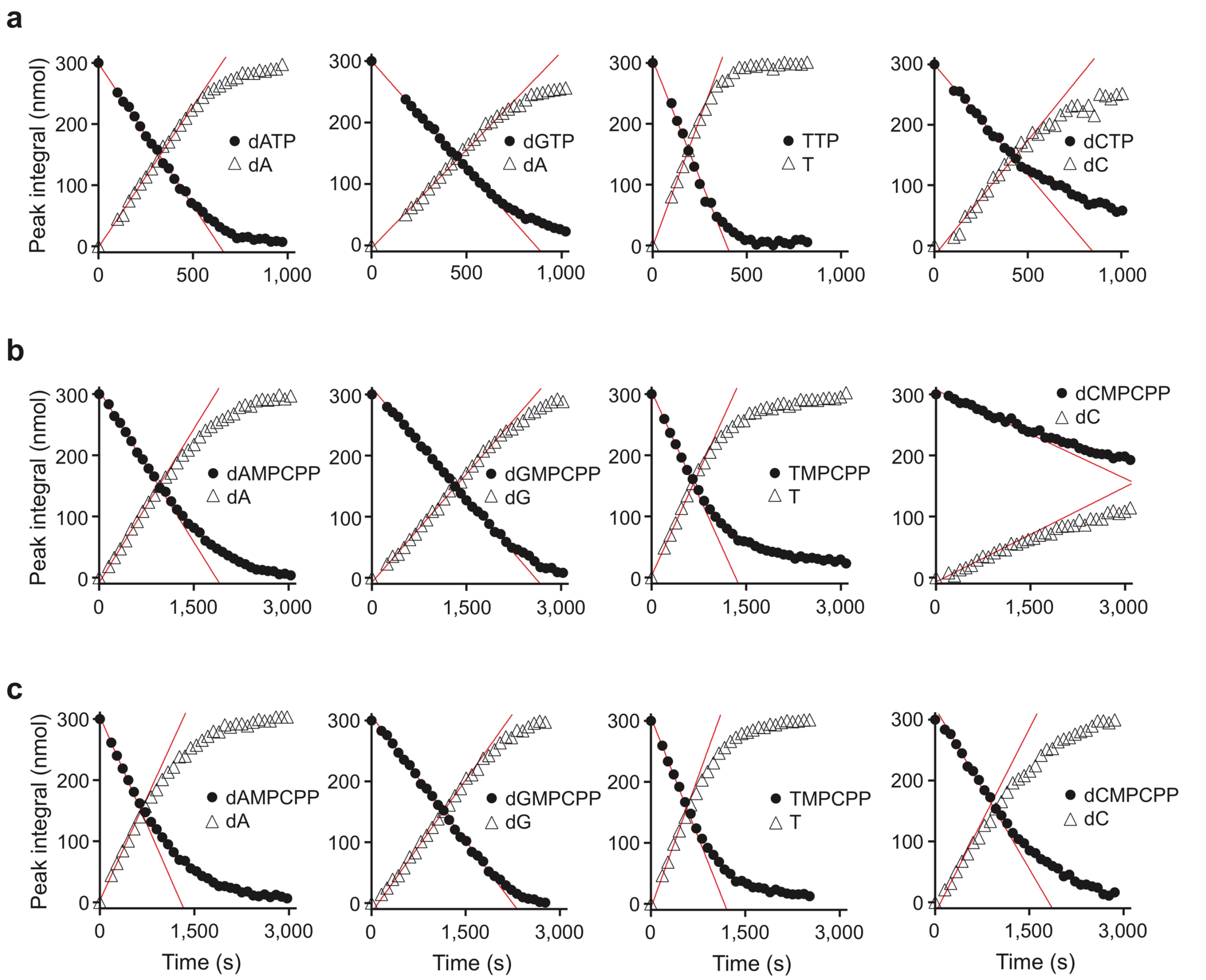

Supplementary Fig. 3. ^{1}H NMR analysis of SAMHD1 hydrolysis of dNMPCPP nucleotides. a, ^{1}H NMR Data recorded for SAMHD1 hydrolysis reactions containing 1 µM SAMHD1, 0.2 mM GTP AL1-activator and 0.5 mM of deoxynucleotides dATP, dGTP, TTP or dCTP. **b**, Data recorded for reactions containing 1 µM SAMHD1, 0.2 mM GTP AL1-activator and 0.5 mM of dAMPCPP, dGMPCPP, TMPCPP or dCMPCPP. **c**, Data recorded for reactions containing 1 µM SAMHD1, 0.2 mM GTP activator, 0.01 mM dATP AL2-activator and 0.5 mM dAMPCPP, dGMPCPP, TMPCPP or dCMPCPP. In each panel, the integral of resolved substrate and product peak resonances from each deoxynucleoside is plotted against time. Initial rates of hydrolysis were determined from slopes (red lines) derived from the data measured in the linear phase of the reaction.

a

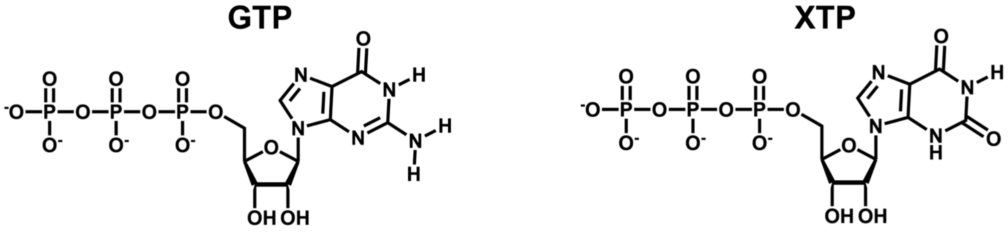

b

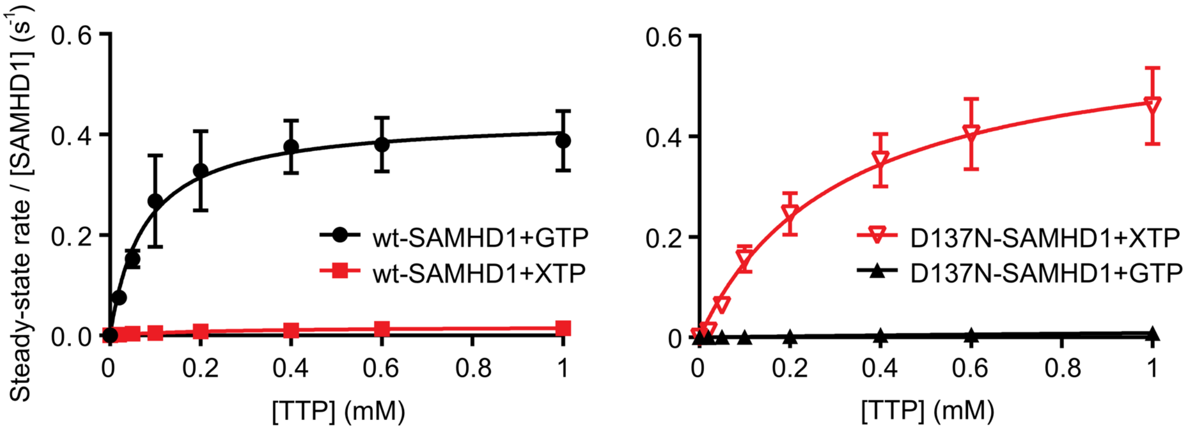

c

SAMHD1 mutant	AL1 activator	K_M (mM)	k_{cat} (s^{-1})	Specificity constant (s^{-1} M^{-1})	Ratio specificity constants (GTP:XTP)
**wt**	**GTP**	0.08±0.01	0.43±0.02	5.38 x 10^3	77:1
**wt**	**XTP**	0.27±0.05	0.02±0.001	70	
**D137N**	**GTP**	>1*	>0.01*	10	1:200
**D137N**	**XTP**	0.30±0.06	0.61±0.04	2 x 10^3	

Supplementary Fig. 4. AL1 specificity and catalytic activity of SAMHD1(D137N). a, Diagrammatic representations of the chemical structures of GTP and XTP. **b**, Comparison of the steady-state kinetics of GTP- and XTP-stimulated hydrolysis of TTP by wt-SAMHD1 and SAMHD1(D137N) using the SAMHD1-Ppx1 continuous coupled enzyme assay. The dependence of the rate on substrate concentration for wt-SAMHD1 (left) and the D137N-SAMHD1 (right) are plotted. The solid lines, black (GTP-stimulated) and red (XTP-stimulated) are the best fit to the data using the Michaelis-Menten expression. Error bars represent the standard deviation of at least three independent measurements. **c**, Steady-state kinetic parameters derived from the data in **b**, values marked with

asterisks are minimum estimates from data fitting. The ratio of specificity constants is the fold increase or decrease in the catalytic specificity constants for GTP relative to XTP for the wt and D137N proteins. Source data for **b** and **c** are provided in the Source Data file.

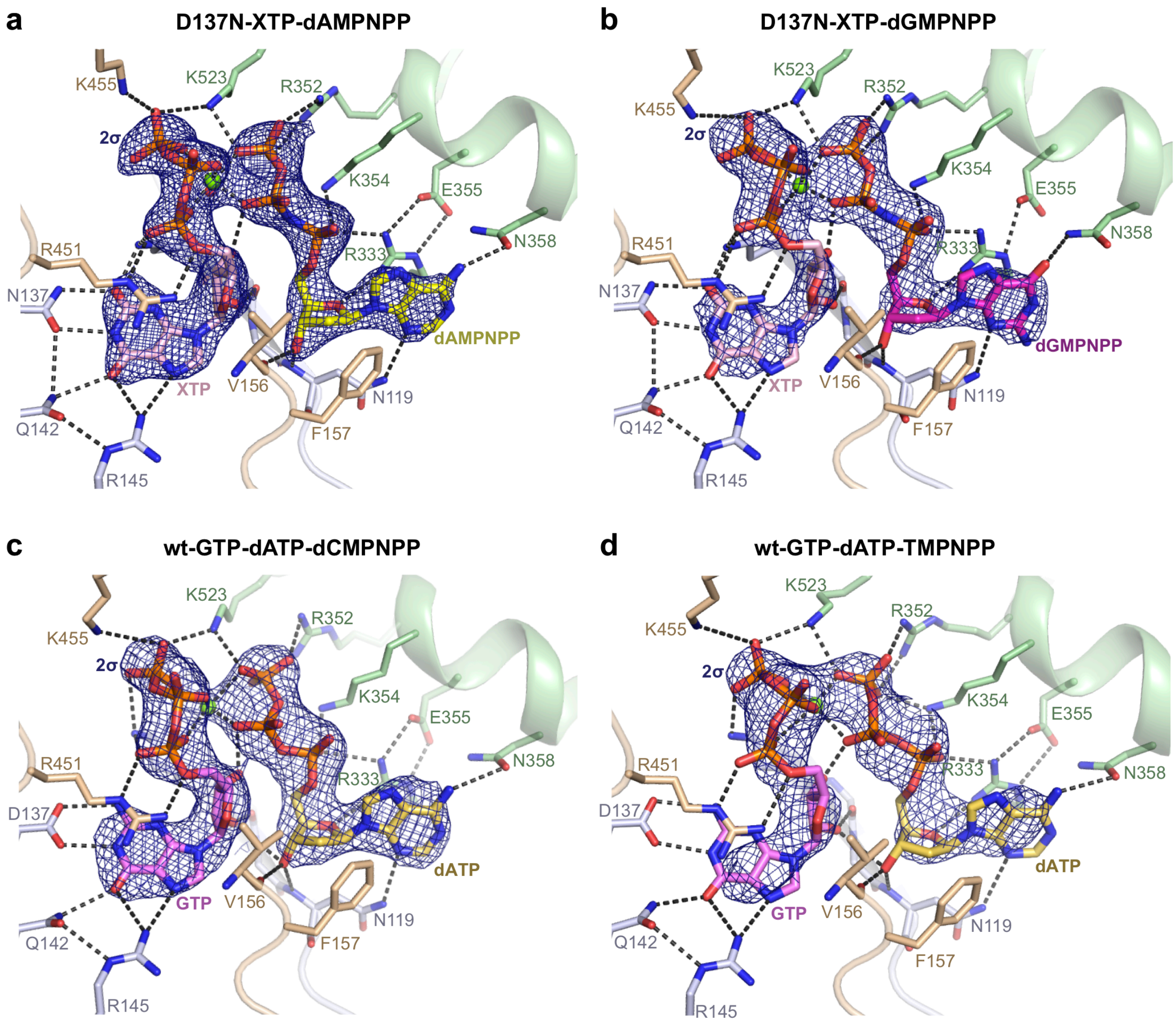

Supplementary Fig. 5. Density for metal ions and nucleotides bound at the allosteric sites. 2Fo – Fc electron density for allosteric site bound nucleotides in SAMHD1 crystal structures. (**a**) D137N-XTP-dAMPNPP, (**b**) D137N-XTP-dGMPNPP, (**c**) wt-GTP-dATP-dCMPNPP and (**d**) wt-GTP-dATP-TMPNPP. The protein backbone is shown in cartoon and the 2Fo-Fc simulated annealing composite omit map is shown as a blue mesh, contoured at 2.0σ. Nucleotides and residues surrounding AL1 and AL2 are shown in stick representation, Mg ions are shown as green spheres. Hydrogen and co-ordinate bonds are displayed as dashed grey lines.

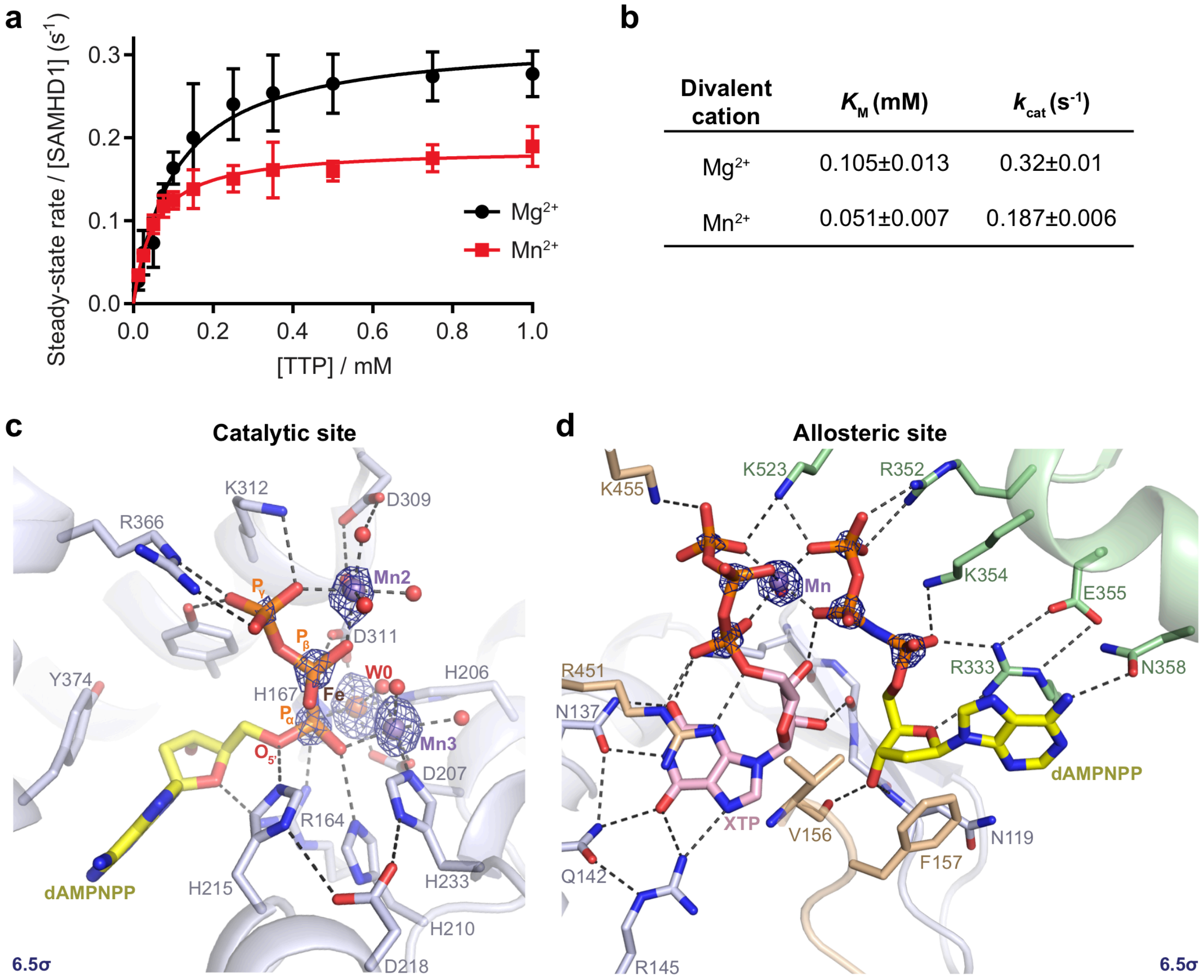

Supplementary Fig. 6. Mg^{2+}- and Mn^{2+}-activated SAMHD1 dNTP hydrolysis. a, Comparison of the steady-state kinetics of GTP-stimulated hydrolysis of TTP by wt-SAMHD1 in the presence of either Mg^{2+} or Mn^{2+} divalent cations. Enzyme activity was measured using the SAMHD1-Ppx1 continuous coupled enzyme assay. The dependence of the rate on substrate concentration for Mg^{2+}-stimulated (black) and Mn^{2+}-stimulated (red) are plotted. The solid lines are the best fit to the data using the Michaelis-Menten expression. Error bars represent the standard deviation of at least two independent measurements. **b**, Steady-state kinetic parameters K_M and k_{cat} derived from the data in **a,** source data for **a** and **b** are provided in the Source Data file. **c & d**, The regions surrounding the active (**c**) and (**d**) allosteric sites are shown for the D137N-SAMHD1(109-626)-XTP-dAMPNPP complex crystallised in the presence of 5 mM $MnCl_2$ with the 2Fo-Fc simulated annealing composite

omit map contoured at 6.5σ shown as blue mesh. Metal ions are shown as spheres coloured by atom type (Fe, brown; Mn, purple), the protein backbone is shown in cartoon representation, bound nucleotides and surrounding residues are shown as sticks and hydrogen and co-ordinate bonds as dashed lines.

a

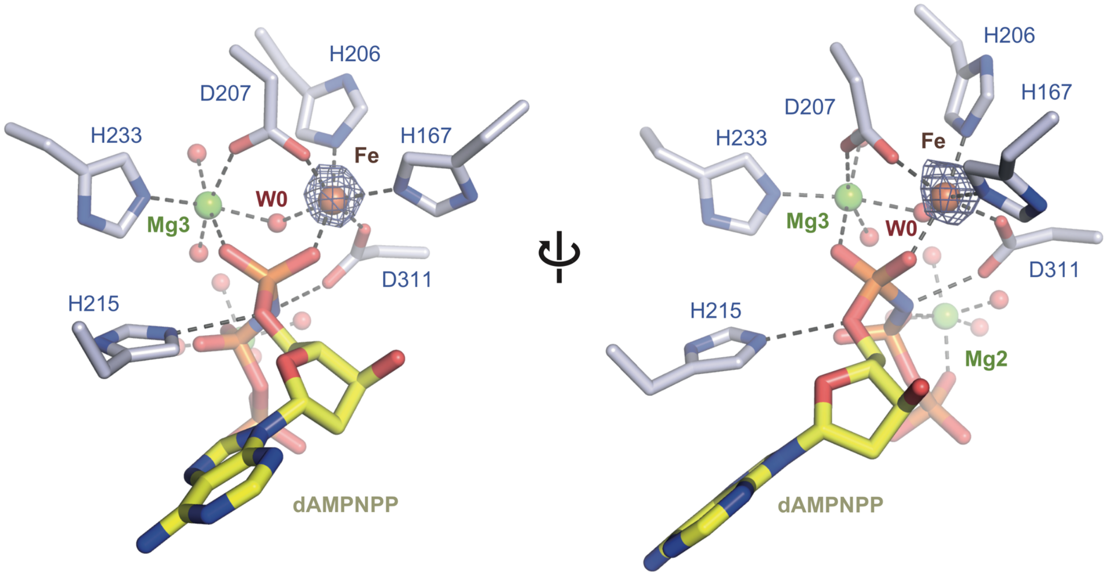

b

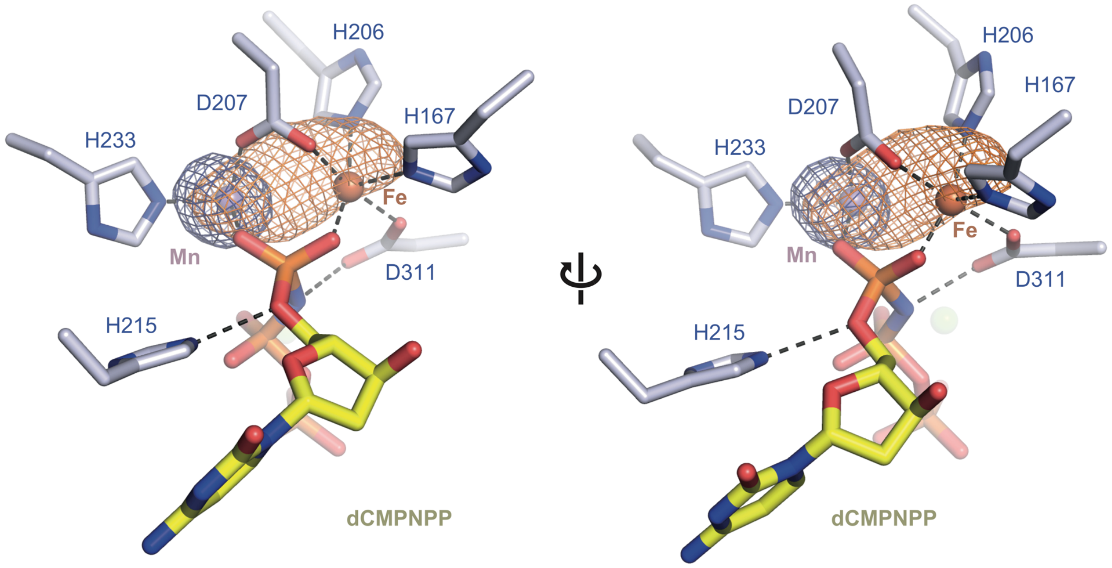

Supplementary Fig. 7. Anomalous scattering of active site metal ions. a, View of the bi-metallic centre in the active site of the D137N-XTP-dAMPNPP-Mg inhibitor complex. The anomalous difference electron density calculated from a dataset recorded at 0.93 Å is shown as blue mesh, contoured at 4 σ. **b**, Anomalous difference density for active site metals recorded from crystals of a D137N-XTP-dATP-dCMPNPP-Mn-Mg inhibitor complex. The anomalous difference density calculated from datasets recorded at the Mn 1.8929 Å and Fe 1.7404 Å absorbance edges is shown

as blue and brown mesh respectively, contoured at 8 σ and 2.2 σ respectively. The views in **a** and **b** are the same orientation as in **Fig. 4a**. Nucleotides and co-ordinating side chains are shown in stick representation with metal ions and co-ordinated water molecules are shown as spheres, coloured by atom type.

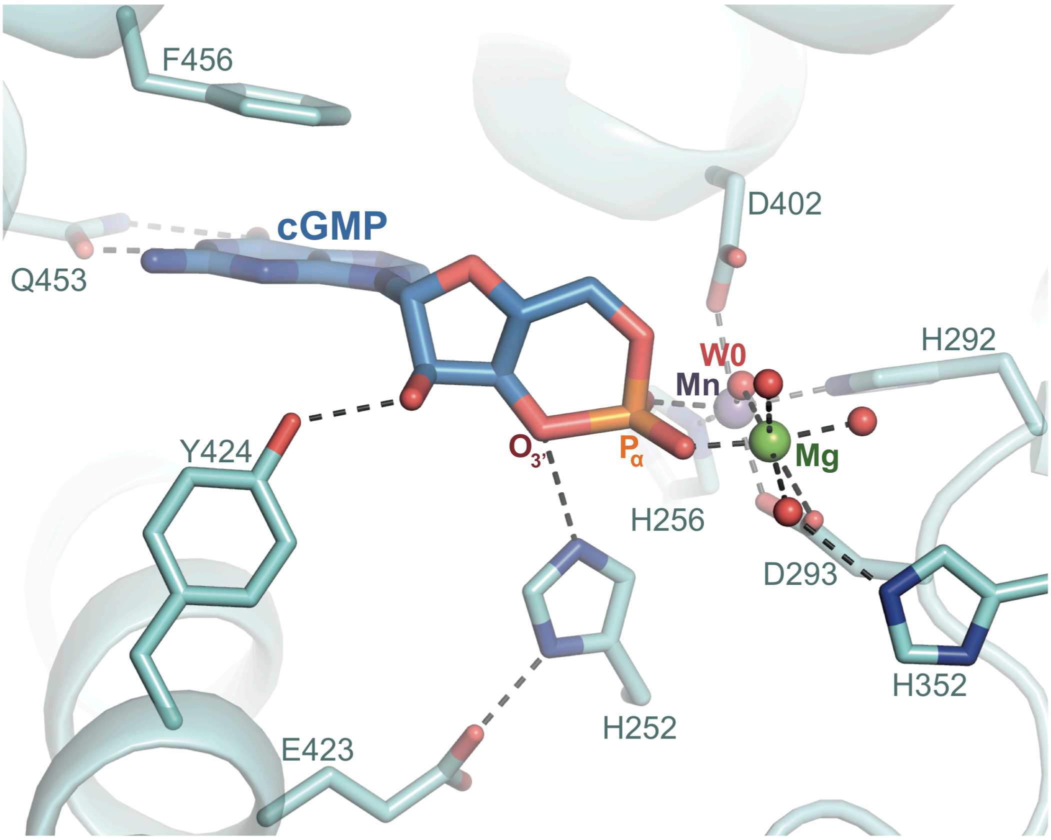

Supplementary Fig. 8. Human PDE9 active site. Close up view of the active site of human PDE9-cGMP complex (PDB ID: 3DYL). The protein backbone is shown in cartoon representation, metal ions and water molecules as spheres. The bound cGMP nucleotide substrate and residues that make interactions with the substrate and active site metal ions are shown as sticks. Hydrogen and co-ordinate bonds are displayed as dashes. W0 is the in-line attacking nucleophilic water. His252 and Glu423 in PDE9 are equivalent to His215 and Asp218 in SAMHD1 which we propose act in a proton relay as a general acid to protonate the $O^{5'}$ of the nucleoside leaving group.

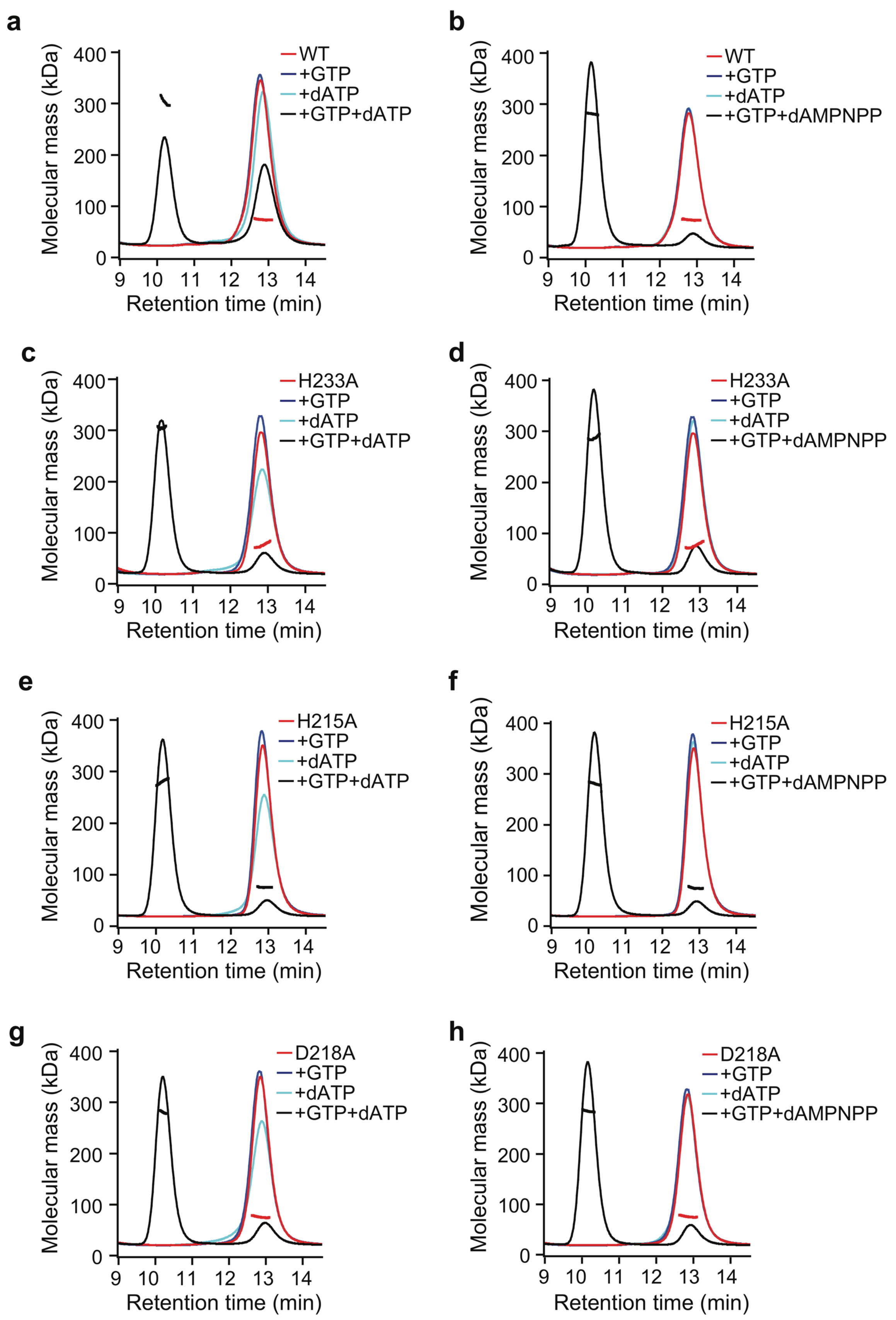

Supplementary Fig. 9. Tetramerisation of active site mutants. SEC-MALLS analysis of monomer-dimer-tetramer equilibrium of wt-SAMHD1 (**a & b**) and active site mutants H233A (**c &d**), H215A (**e**

& f) and D218A (**g & h**) upon addition of dATP and dAMPNPP nucleotides, respectively. In each panel, the solid lines are the chromatograms from the output of the differential refractometer and the black scatter points are the weight-averaged molar masses determined at 1-second intervals throughout elution of chromatographic peaks. The curves shown are: (red) apo-SAMHD1; (blue) SAMHD1 and 0.2 mM GTP; (cyan) SAMHD1 and 0.5 mM indicated dATP or dAMPNPP; and (black) SAMHD1, 0.2 mM GTP and 0.5 mM indicated dATP or dAMPNPP. All mutants induce SAMHD1 tetramerisation, despite catalytic deficiency.

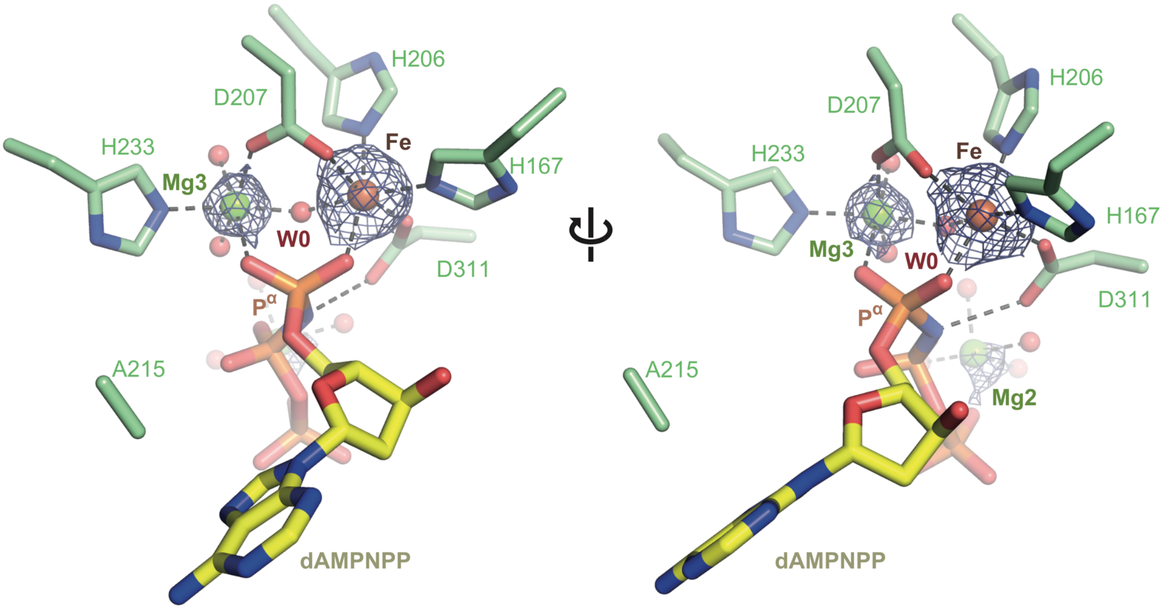

Supplementary Fig. 10. Active site of H215A-SAMHD1(109-626)-GTP-dAMPNPP inhibitor complex. View of the bi-metallic centre in the active site of the H215A-SAMHD1(109-626)-GTP-dAMPNPP inhibitor complex with electron density for bound metal ions. Nucleotides are shown in stick representation. Metal ions and co-ordinated water molecules are shown as spheres, coloured by atom type as in **Fig 6c**. Hydrogen bonding and metal ion co-ordinate bonds are shown as dashed lines. The electron density (blue mesh) for the Fe, Mg2 and Mg3 ions is the simulated-annealing composite omit 2Fo – Fc map, contoured at 3.3 σ.

Supplementary Table 1. dNMPNPP inhibition of SAMHD1

dNTP analogue	AL1 Activator	Substrate	K_i (μM)
dAMPNPP	GTP	TTP	0.78 ± 0.06
dGMPNPP	GTP/dGMPNPP	TTP	0.052 ± 0.002
TMPNPP	GTP	TTP	0.37 ± 0.02
dCMPNPP	GTP	TTP	0.073 ± 0.003

Supplementary Table 2. Catalytic turnover of dNTPs and dNMPCPP analogues

Substrate	AL2 Activator	AL1 Activator	k_{cat} (s^{-1})
dATP	dATP	GTP	0.86±0.09
dGTP	dGTP	GTP / dGTP	0.66±0.15
TTP	TTP	GTP	1.43±0.07
dCTP	dCTP	GTP	0.57±0.11
dAMPCPP	dAMPCPP	GTP	0.21±0.09
dGMPCPP	dGMPCPP	GTP / dGMPCPP	0.26±0.08
TMPCPP	TMPCPP	GTP	0.38±0.01
dCMPCPP	dCMPCPP	GTP	0.075±0.008
dAMPCPP	dAMPCPP / dATP*	GTP	0.35±0.04
dGMPCPP	dGMPCPP / dATP*	GTP / dGMPCPP	0.23±0.04
TMPCPP	TMPCPP / dATP*	GTP	0.44±0.09
dCMPCPP	dCMPCPP / dATP*	GTP	0.21±0.06

*Reactions were supplemented with 10 μM dATP as an additional AL2 activator

Supplementary Table 3. X-ray data collection and structure refinement statistics

	D137N-SAMHD1 Mg, XTP, dAMPNPP	D137N-SAMHD1 Mg, XTP, dAMPNPP (STARANISO)	D137N-SAMHD1 Mg, XTP, dGMPNPP	D137N-SAMHD1 Mg, XTP, dGMPNPP (STARANISO)
**Data collection**				
Space group	P 4_3 2_1 2	P 4_3 2_1 2	P 2_1	P 2_1
Cell dimensions				
a, b, c (Å)	105.9, 105.9, 198.1	105.9, 105.9, 198.1	97.8, 173.0, 276.0	97.8, 172.9, 275.9
α, β, γ	90, 90, 90	90, 90, 90	90, 95.2, 90	90, 95.2, 90
Wavelength (Å)	0.92819	0.92819	0.96861	0.96861
Resolution (Å)	72.32(2.44)-2.40	72.34(2.26)-2.01	137.41(3.36)-3.30	146.33(3.20)-2.85
Anisotropic diff. limits				
a*, b*, c* (Å)	-	2.57, 2.57, 1.98	-	3.44, 3.39, 2.84
Unique reflections	44,919 (2,202)[†]	45,483 (2,274)	137,345 (6,691)	140,978 (7,049)
R_{meas} (%)	11.9 (123)	12.0 (126)	20.2 (61.2)	22.0 (68)
R_{pim} (%)	3.9 (42)	3.9 (42)	10.8 (32.8)	11.7 (36)
$CC_{1/2}$	0.999 (0.790)	0.999 (0.674)	0.948 (0.765)	0.980 (0.729)
I/δ(I)	11.4 (1.7)	14.3 (2.0)	5.8 (2.3)	4.0 (1.5)
Completeness				
Spherical (%)	100 (100)	59.8 (10.2)	99.77 (97.34)	66.5 (11.4)
Ellipsoidal (%)	-	95.6 (78.8)	-	94.3 (67.3)
Multiplicity	9.1 (8.6)	9.3 (8.9)	3.4 (3.4)	3.5 (3.4)
**Refinement**				
Resolution (Å)		72.31-2.01		146.24-2.85
R_{work}/R_{free}/Test set (%)		18.36 / 22.81 / 5.07		19.44 / 21.18 / 4.98
No. monomers/A.S.U.		2		16
No. atoms				
Protein		7,720		61,333
Nucleotide		184		1,504
Ligand Fe		2		16
Ligand Mg		6		48
Ligand Mn		0		0
Ligand SO4		0		75
Water		128		0
B-factors (Å)				
Wilson		36.1		49.5
Protein		44.9		71.0
Nucleotide		34.4		43.7
Ligand Fe		32.5		42.1
Ligand Mg		41.4		27.2
Ligand Mn		-		-
Ligand SO4		-		112.7
Water		35.3		-
Average		44.4		70.4
R.m.s. deviations				
Bond lengths (Å)		0.0202		0.0110
Bond angles (°)		2.0239		1.5351
Chiral volumes ($Å^3$)		0.1148		0.0848
Ramachandran				
Favoured		911 (96.0%)		7,364 (96.8%)
Allowed		37 (3.9%)		238 (3.1%)
Outliers		1 (0.1%)		2 (0.03%)
PDB code		6TX0		6TXA

Supplementary Table 3 - continued

	wt-SAMHD1 Mg, GTP, dATP dCMPNPP	wt-SAMHD1 Mg, GTP, dATP, dCMPNPP (STARANISO)	wt-SAMHD1 Mg, GTP, dATP, dTMPNPP	wt-SAMHD1 Mg, GTP, dATP, dTMPNPP (STARANISO)
**Data collection**				
Space group	$P\ 2_1$	$P\ 2_1$	$P\ 2_1$	$P\ 2_1$
Cell dimensions				
a, b, c (Å)	99.3, 175.6, 277.7	99.1, 175.2, 277.3	97.5, 172.2, 275.2	97.5, 172.2, 275.3
α, β, γ	90, 94.9, 90	90, 94.8, 90	90, 95.3, 90	90, 95.3, 90
Wavelength (Å)	0.97625	0.97625	0.97950	0.97950
Resolution (Å)	98.95(3.25)-3.19	276.29(3.10)-2.84	88.93(3.76)-3.70	91.36(3.63)-3.19
Anisotropic diff. limits				
a*, b*, c* (Å)	-	2.82, 3.05, 3.48	-	3.90, 4.60, 3.18
Unique reflections	156,298 (7,098)	161,935 (8,099)	96,051 (4,812)	80,315 (4,016)
R_{meas} (%)	17.1 (66)	19.5 (102)	38.7 (113)	46.7 (126)
R_{pim} (%)	8.6 (33)	9.7 (49)	14.7 (43)	17.5 (47.6)
$CC_{1/2}$	0.843 (0.824)	0.991 (0.595)	0.972 (0.818)	0.960 (0.668)
I/δ(I)	6.3 (2.0)	7.1 (1.5)	4.8 (1.8)	4.7 (1.7)
Completeness			-	
Spherical (%)	99.5 (91.5)	72.9 (15.8)	99.5 (98.8)	53.2 (8.2)
Ellipsoidal (%)	-	93.7 (53.8)	-	92.3 (76.2)
Multiplicity	3.7 (3.7)	3.9 (4.1)	6.8 (6.8)	7.0 (6.7)
**Refinement**				
Resolution (Å)		276.29-2.84		91.22-3.19
R_{work}/R_{free}/Test set (%)		22.80 / 24.89 / 4.98		21.79 / 25.18 / 5.00
No. monomers/A.S.U.		16		16
No. atoms				
Protein		60,407		61,116
Nucleotide		1,440		1,456
Ligand Fe		16		16
Ligand Mg		42		32
Ligand Mn		0		0
Ligand SO4		45		55
Water		0		0
B-factors (Å)				
Wilson		46.2		50.1
Protein		54.6		85.1
Nucleotide		59.8		65.9
Ligand Fe		49.0		47.9
Ligand Mg		57.9		42.4
Ligand Mn		-		-
Ligand SO4		112.7		118.8
Water		-		-
Average		54.3		84.4
R.m.s. deviations				
Bond lengths (Å)		0.0132		0.0095
Bond angles (°)		1.7023		1.4111
Chiral volumes ($Å^3$)		0.0874		0.0729
Ramachandran				
Favoured		7,272 (96.8%)		7,331 (96.5%)
Allowed		238 (3.2%)		260 (3.4%)
Outliers		3 (0.04%)		1 (0.01%)
PDB code		6TXC		6TXE

Supplementary Table 3 - continued

	D137N-SAMHD1 Mn, XTP, dAMPNPP	D137N-SAMHD1 Mn, Mg, XTP, dATP dCMPNPP	H215A-SAMHD1 Mg, GTP, dAMPNPP
**Data collection**			
Space group	$P\ 4_3\ 2_1\ 2$	$P\ 4_3\ 2_1\ 2$	$P\ 2_1\ 2_1\ 2_1$
Cell dimensions			
a, b, c (Å)	105.0, 105.0, 195.5	105.3, 105.3, 195.6	137.4, 171.9, 179.6
α, β, γ	90, 90, 90	90, 90, 90	90, 90, 90
Wavelength (Å)	0.97866	1.9075	0.97624
Resolution (Å)	71.54(2.29)-2.25	195.6(3.31)-3.25	107.30(2.24)-2.20
Anisotropic diff. limits			-
a*, b*, c* (Å)	-	-	-
Unique reflections	51,603 (2,085)	18,094 (885)	212,120 (8,698)
R_{meas} (%)	12.6 (131)	20.4 (134)	19.7 (118)
R_{pim} (%)	2.9 (46.0)	5.3 (34.9)	7.4 (52.5)
$CC_{1/2}$	0.998 (0.673)	0.997 (0.752)	0.988 (0.517)
I/δ(I)	14.5 (1.8)	10.5 (2.1)	8.2 (1.5)
Completeness			
Spherical (%)	97.75 (80.13)	100.00 (99.44)	98.7 (81.7)
Ellipsoidal (%)	-	-	-
Multiplicity	16.8 (6.8)	15.4 (15.4)	6.9 (4.8)
**Refinement**			
Resolution (Å)	71.51-2.25	92.72-3.25	107.30-2.20
R_{work}/R_{free}/Test set (%)	19.52 / 23.88 / 5.01	17.59 / 23.59 / 5.16	17.64 / 20.24 / 4.96
No. monomers/A.S.U.	2	2	8
No. atoms			
Protein	7,748	7,210	30,873
Nucleotide	184	188	736
Ligand Fe	2	2	8
Ligand Mg	0	4	25
Ligand Mn	6	2	0
Ligand SO4	0	0	0
Water	90	0	796
B-factors (Å)			
Wilson	37.6	86.2	24.4
Protein	48.5	109.6	31.5
Nucleotide	43.5	77.8	23.2
Ligand Fe	41.4	68.6	20.2
Ligand Mg	-	66.9	23.4
Ligand Mn	49.0	75.0	-
Ligand SO4	-	-	-
Water	32.8	-	23.7
Average	47.9	108.8	31.0
R.m.s. deviations			
Bond lengths (Å)	0.0211	0.0146	0.0211
Bond angles (°)	2.0953	1.8646	2.0922
Chiral volumes (Å^3)	0.1222	0.0974	0.1256
Ramachandran			
Favoured	928 (97.7%)	864 (93.5%)	3,718 (98.3%)
Allowed	22 (2.3%)	55 (6.0%)	66 (1.7%)
Outliers	0 (0%)	5 (0.5%)	0 (0%)
PDB code	6TXF	6YOM	6XU1

†Values in parentheses refer to the highest resolution shell

Supplementary Table 4 nucleotide and metal contents of SAMHD1 structures

Structure	AL1 (nuc)	AL2 (nuc)	AL1/AL2 (metal)	Active site (nuc)	Active site (metal)
D137N-SAMHD1, Mg, XTP, dAMPNPP	XTP	dAMPNPP	Mg	dAMPNPP	Fe, Mg
D137N-SAMHD1, Mg, XTP, dGMPNPP	XTP	dGMPNPP	Mg	dGMPPNPP	Fe, Mg
wt-SAMHD1, Mg, GTP, dATP, dCMPNPP	GTP	dATP	Mg	dCMPNPP	Fe, Mg
wt-SAMHD1, Mg, GTP, dATP, TMPNPP	GTP	dATP	Mg	TMPNPP	Fe, Mg
D137N-SAMHD1, Mn, XTP, dAMPNPP	XTP	dAMPNPP	Mn	dAMPNPP	Fe, Mn
D137N-SAMHD1, Mn, Mg, XTP, dATP, dCMPNPP	XTP	dATP	Mg	dCMPNPP	Fe, Mn, Mg
H215A-SAMHD1, Mg, GTP, dAMPNPP	GTP	dAMPNPP	Mg	dAMPNPP	Fe, Mg

Supplementary Table 5. Primers for SAMHD1 cloning and mutagenesis

Construct		Primers (5' - 3')*#
M1-M626 (full length)	FWD	CAGGGACCCGGTATGCAGCGAGCCGATTCCGAGCAG
	REV	GGCACCAGAGCGTTACATTGGGTCATCTTTAAAAAGCTG
Q109-M626	FWD	GGCCCCGGGCAAATCCACGTTGATACAATG
	REV	GGCGCGGCCGCTCATCACATTGGGTCATCTTTAAAAAGCTGG
D137N	FWD	CTCGTCCGAATCATTAATACACCTCAATTTCAAC
	REV	GTTGAAATTGAGGTGTATTAATGATTCGGACGAG
H215A	FWD	GTCATGGGCCATTTTCTGCCATGTTTGATGGACGATTTATTC
	REV	GAATAAATCGTCCATCAAACATGGCAGAAAATGGCCCATGAC
H233A	FWD	GGAGGTGAAATGGACGGCTGAACAAGGCTCAGTTATG
	REV	CATAACTGAGCCTTGTTCAGCCGTCCATTTCACCTCC
D218A	FWD	CCATTTTCTCACATGTTTGCTGGACGATTTATTCCACTTG
	REV	CAAGTGGAATAAATCGTCCAGCAAACATGTGAGAAAATGG

*LIC or restriction sites used for cloning are underlined
#Mutagenized codons are highlighted and underlined
